# *In vivo* analysis of hybrid hydrogels containing dual growth factor combinations, and skeletal stem cells under mechanical stimulation for bone repair

**DOI:** 10.1016/j.mbm.2024.100096

**Published:** 2024-08-27

**Authors:** David Gothard, Michael Rotherham, Emma L. Smith, Janos M. Kanczler, James Henstock, Julia A. Wells, Carol A. Roberts, Omar Qutachi, Heather Peto, Hassan Rashidi, Luis Rojo, Lisa J. White, Molly M. Stevens, Alicia J. El Haj, Felicity R.A.J. Rose, Richard O.C. Oreffo

**Affiliations:** a Bone and Joint Research Group, Centre for Human Development, Stem Cells and Regeneration, Institute of Developmental Sciences, University of Southampton, Southampton, SO16 6YD, UK; b Institute for Science and Technology in Medicine, Keele University, Guy Hilton Research Centre, Stoke-on-Trent, ST4 7BQ, UK; c Healthcare Technologies Institute, School of Chemical Engineering, Institute of Translational Medicine, University of Birmingham, Birmingham, B15 2TH, UK; d Department of Applied Sciences, Pandon Building, Northumbria University, Newcastle-upon-Tyne, NE2 1XE, UK; e Wolfson Centre for Stem Cells, Tissue Engineering and Modelling, University of Nottingham, Centre for Biomolecular Sciences, University Park, Nottingham, NG7 2RD, UK; f Leicester Institute for Pharmaceutical Innovation (LIPI), Leicester School of Pharmacy, Faculty of Health and Life Sciences, De Montfort University, The Gateway, Leicester, LE1 9BH, United Kingdom; g Stem Cells and Regenerative Medicine Section, UCL Great Ormond Street Institute of Child Health, University College London, London, WC1N 1EH, UK; h Department of Materials, Imperial College London, Royal School of Mines, London, SW7 2AZ, UK; i Department of Bioengineering, Imperial College London, South Kensington Campus, London, SW7 2AZ, UK; j Instituto de Ciencia y Tecnología de Polímeros (ICTP), CSIC, Calle Juan de la Cierva, 3, 28006 Madrid, Spain; k Consorcio Centro de Investigación Biomédica en Red, CIBER-BBN, Instituto de Salud Carlos III, Calle Monforte de Lemos 3–5, 1128029 Pabellón, Madrid, Spain; l Nottingham Biodiscovery Institute, School of Pharmacy, University of Nottingham, Nottingham, NG7 2RD, UK; m Institute for Biomedical Engineering Imperial College London, South Kensington Campus, London, SW7 2AZ, UK; n Department of Physiology, Anatomy, & Genetics, Department of Engineering Science, and Kavli Institute for Nanoscience Discovery, University of Oxford, Oxford, OX1 3QU, UK

**Keywords:** Growth factors, Controlled release, Bone ECM, Bone formation, Mechanotransduction, Skeletal stem cell

## Abstract

Bone tissue engineering requires a combination of materials, cells, growth factors and mechanical cues to recapitulate bone formation. In this study we evaluated hybrid hydrogels for minimally invasive bone formation by combining biomaterials with skeletal stem cells and staged release of growth factors together with mechanotransduction. Hybrid hydrogels consisting of alginate and decellularized, demineralised bone extracellular matrix (ALG/ECM) were seeded with Stro-1+ human bone marrow stromal cells (HBMSCs). Dual combinations of growth factors within staged-release polylactic-co-glycolic acid (PLGA) microparticles were added to hydrogels to mimic, in part, the signalling events in bone regeneration: VEGF, TGF-β_3,_ PTHrP (fast release), or BMP-2, vitamin D_3_ (slow release). Mechanotransduction was initiated using magnetic fields to remotely actuate superparamagnetic nanoparticles (MNP) targeted to TREK1 ion channels. Hybrid hydrogels were implanted subcutaneously within mice for 28 days, and evaluated for bone formation using micro-CT and histology. Control hydrogels lacking HBMSCs, growth factors, or MNP became mineralised, and neither growth factors, HBMSCs, nor mechanotransduction increased bone formation. However, structural differences in the newly-formed bone were influenced by growth factors. Slow release of BMP-2 induced thick bone trabeculae and PTHrP or VitD_3_ increased bone formation. However, fast-release of TGF-β_3_ and VEGF resulted in thin trabeculae. Mechanotransduction reversed the trabecular thinning and increased collagen deposition with PTHrP and VitD_3_. Our findings demonstrate the potential of hybrid ALG/ECM hydrogel–cell–growth factor constructs to repair bone in combination with mechanotransduction for fine-tuning bone structure. This approach may form a minimally invasive reparative strategy for bone tissue engineering applications.

## 1 Introduction

Tissue engineering is an important component of regenerative medicine and heralds a change from sourcing compatible donor tissues, to creating required tissues on demand.^1^ The current ‘gold standard’ treatment for skeletal tissue replacement sources auto- and allogenic bone grafts. However, there are limitations to their clinical use including availability, complex preparation procedures, and donor variability affecting regenerative capacity. Consequently, alternative approaches to generate tissue engineered constructs are required.^2^, ^3^, ^4^, ^5^, ^6^

Bone tissue engineering typically harnesses skeletal stem cells (SSCs), growth factors, biomaterials, and physico-chemical induction. SSCs provide a renewable and inducible cell source with which to repopulate a defect site, while physico-chemical induction is important to provide sufficient and appropriate guidance for SSC proliferation, colonisation, and differentiation within a tissue engineered construct and environment.^7^^,^^8^ Indeed, spatiotemporal delivery of inductive signals such as growth factors can be achieved through encapsulation within polymeric biomaterial scaffolds or controlled release from microparticles for localised inductive signalling.^9^, ^10^, ^11^, ^12^ Careful spatiotemporal delivery of select growth factors is paramount to any successful tissue engineering strategy. There are a number of signalling factors which are critical in regulating endochondral bone formation, principal factors include Transforming growth factor β3 (TGF-β3), which promotes chondrogenic differentiation of SSC and formation of cartilage. While growth factors such as Parathyroid hormone-related protein (PTHrP), Bone morphogenetic protein 2 (BMP-2) and vitamin D_3_ (VitD_3_) are known osteoinductive factors pivotal in the endochondral ossification process. In addition to chondrogenic and osteogenic differentiation factors, the role of vascularisation is critical to the growth of new bone formation, in this respect Vascular endothelial growth factor (VEGF) provides important signalling cues for blood vessel development in new bone formation.^13^ As well as biochemical cues, physical stimulation, or mechanotransduction, play important roles in normal bone formation. However, mechanotransduction and the role of physical cues are often overlooked in many tissue engineering regimens. Mechanotransduction has been shown to be an integral part of the osteoblast differentiation process, acting in concert with growth factor induced signalling cascades.^14^, ^15^, ^16^ Mechanotransduction is an important process in regulating bone tissue formation and is initiated *in vivo* by external forces such fluid shear and pressure.^17^ Mechanotransduction can be replicated *in vitro* using bioreactor systems to impart shear and compressive forces to cells and tissues, which has been shown to enhance bone formation *in vitro*.^18^^,^^19^ We have previously demonstrated a role for remote stimulation of mechanotransduction to enhance skeletal cell function using a biomagnetic approach, where functionalised superparamagnetic iron oxide nanoparticles (MNP) are tagged to cell-surface mechanoreceptors. The particle bound receptors are activated by oscillating the particles in a magnetic field which provides direct mechanical forces to the receptors which induces downstream signalling cascades and promotes differentiation.^20^, ^21^, ^22^ Induction of mechanotransduction in this approach confers advantages over alternative force generating bioreactors for bone tissue engineering as the force applied by magnetic particles is tuneable. Furthermore, the approach is amenable as a temporal, non-invasive injectable therapy and signalling can be remotely controlled externally.^21^

Successful tissue engineering approaches are crucially dependent on biomaterials that exhibit properties compatible with the host tissue, with hydrogels being widely used within the tissue engineering field..^23^, ^24^, ^25^ Hydrogels are polymeric networks comprising natural and/or synthetic polymers^26^^,^^27^ and have been utilised in a number of regenerative approaches, often with growth factor incorporation to provide cues for effective differentiation of host stem cells, for bone tissue engineering.^28^, ^29^, ^30^

The current studies have utilised a hybrid bone ECM-derived hydrogel as an osteoconductive and osteoinductive scaffold support for the delivery of SSCs and select angio-, chondro-, and osteogenic growth factors to a bone defect site under mechanical stimulation. Based on previous work by Sawkins and colleagues,^31^ purified bone ECM hydrogel offers a biomaterial scaffold that is predominantly composed of macromolecules that are highly conserved across species, and helps to reduce any potential immunogenic and inflammatory complications.^32^^,^^33^ Hydrogel constructs in the present study were prepared as previously described.^34^ Briefly, bone ECM was mixed with alginate, Stro-1-enriched (Stro-1+) human bone marrow stromal cells (HBMSCs) and growth factor-loaded poly(D,L-lactic-co-glycolic acid) (P_DL_LGA) microparticles. An ‘in-house’ triblock polymer P_DL_LGA-poly (ethylene glycol) (PEG)-P_DL_LGA^35^^,^^36^ enabled production of microparticles with two distinct kinetic profiles for fast (days) and slow (weeks) release.^36^ Microparticles were loaded with individual angiogenic (VEGF – fast release), chondrogenic (TGF-β_3_ – fast release), and osteogenic (PTHrP – fast release, BMP-2 or VitD_3_– slow release) growth factors, and human serum albumin (HSA) as a carrier protein. Microparticle combinations were incorporated into the hydrogel constructs to assess the efficacy of dual growth factor delivery; establishing more complex signalling for complex tissue formation.

Previous work within the group assessed single growth factor delivery in a subcutaneous mouse model,^34^ and bone formation within an *in vitro* organotypic culture of chick femur defects with implanted hydrogels.^11^^,^^12^ Mechanotransduction via the mechanosensitive potassium channel TREK1 using TREK targeted MNPs^37^ has previously been shown to induce and augment osteogenic differentiation within HBMSCs and marrow stromal cells.^38^, ^39^, ^40^ Here, hydrogels were assessed *in vivo* with and without additional mechanical stimulation using SSCs immune-labelled with MNPs tethered to the mechanically gated ion channel TREK1. Hydrogel constructs were subsequently implanted subcutaneously within immune-deficient mice for 28 days and exposed to a magnetic field to assess bone formation.

The current study details the bone formation capacity of hybrid alginate/bone ECM (ALG/ECM) hydrogel constructs *in vivo*, predicated on the hypothesis that controlled and phased delivery of dual growth factor combinations would reflect, at least in part, native *in vivo* signalling, leading to improved bone formation compared with the single growth factor delivery regime as previously reported.^34^ Mechanotransduction was implemented to further enhance bone formation, replicating the physical stimulus experienced by cells and tissues within the native environment. This study uniquely combines the central facets of bone tissue engineering; cells, materials, multiple growth factors and mechanical forces, with the aim of assessing the effectiveness of this approach as a bone repair strategy.

## 2 Materials and methods

All materials were purchased from Sigma Aldrich unless otherwise stated.

### 2.1 Ethics statement

Human bone marrow was collected following informed consent (approval from the Southampton and South West Hampshire Local Research Ethics Committee (LREC194/99)) from patients undergoing total hip-replacement surgery. All animal procedures were carried out in accordance with the guidelines and regulations laid down in the Animals (Scientific Procedures) Act 1986. MF1 nu/nu mice were sacrificed after 28 days using schedule 1 CO_2_ inhalation and cervical dislocation according to Home Office Approval UK (Project license – PPL 30/2762). All surgery was performed under anaesthesia/analgesia, and all efforts were made to minimise suffering.

### 2.2 Marrow preparation and cell isolation

Human bone marrow collected from haematologically normal osteoarthritic patients (4 patients, mean age 63 ​± ​19 years standard deviation) was suspended in modified eagle's medium - alpha (α-MEM) and centrifuged at 1000 ​rpm for 4 ​min to remove fat. Blood clots and bone fragments were removed by sieving (40 ​μm pores) before density gradient centrifugation at 2200 ​rpm and 18 ​°C for 40 ​min using LymphoPrep™ (Lonza) to remove red blood cells (RBCs). The buffy layer above the LymphoPrep™ was transferred to a fresh 50 ​mL Falcon tube (BD Bioscience), topped with α-MEM and centrifuged at 1000 ​rpm for 4 ​min. Isolated cell pellets were washed with phosphate buffered saline (PBS, Lonza) and suspended in blocking buffer (α-MEM, 10 % (v/v) human serum, 5 % (v/v) foetal calf serum (FCS) and 10 ​mg/mL bovine serum albumin [BSA] for 15 ​min at 4 ​°C. Cells were incubated with primary antibody (anti-human Stro-1, Hybridoma culture) for 30 ​min and then magnetic bead conjugated secondary antibody (200 ​μL in 800 ​μL isolation buffer, Miltenyi Biotec) for 15 ​min, both at 4 ​°C.^41^ Cells were washed with isolation buffer (0.5 % (w/v) BSA and 2 ​mM ethylenediaminetetraacetic acid (EDTA) in distilled H_2_O [dH_2_O]) between steps. Immuno-labelled cells were subsequently isolated by magnetic-activated cell sorting (MACS) prior to *in vitro* culture expansion in basal media (α-MEM, 10 % (v/v) FCS, penicillin [100 U/mL] and streptomycin [0.1 ​mg/mL]). Cells were cultured at 37 ​°C and 5 % CO_2_ in a humidified atmosphere through two serial passages until sufficient cell numbers were achieved for incorporation within hydrogels. Cells from the 4 donor patients were mixed before use and incorporation within hydrogels.

### 2.3 Surface functionalisation of magnetic nanoparticles

MNPs with a diameter of 250 ​nm and carboxyl functionalisation (Micromod, Germany) were coated with primary antibody (rabbit anti-human TREK1 [Alomone labs] using a carbodiimide activation method. Briefly, MNPs (1 ​mg) were activated in 31 ​mM 1-ethyl-3-(3-dimethylaminopropyl)-carbodiimide hydrochloride (EDAC) and 104 ​mM N-hydroxysuccinimide (NHS) dissolved in 2-(N-Morpholino) ethanesulfonic acid (MES) buffer solution (0.5 ​M, pH adjusted to 6.3), for 1 ​h at room temperature. Activated MNPs were then washed with 0.1 ​M MES buffer on a permanent magnet, re-suspended in secondary antibody solution (20 ​μg goat anti-rabbit IgG [Abcam] diluted in 0.1 ​M MES buffer), and mixed overnight at 4 ​^°^C. MNPs were again washed (on permanent magnet) and re-suspended in 0.1 ​M MES buffer containing primary antibody (10 ​μg) and mixed for 3 ​h at room temperature. Free carboxyl groups were blocked with 100 ​mM glycine for 30 ​min. TREK-coated MNPs (TREK-MNPs) were subsequently washed and re-suspended in 1 ​mL BSA (0.1 % (w/v) in PBS). N-[1-(2,3-dioleoyloxy)propyl]-N,N,N-trimethyl ammonium methyl-sulfate (DOTAP, 40 ​ng/mg MNPs) was added to promote cell surface adhesion and internalisation of TREK-MNPs, prior to cell labelling. Following *in vitro* expansion (P2), washing with PBS, and trypsinisation, Stro-1+cells were suspended in calcium-free Dulbecco's modified eagle's medium (DMEM, Invitrogen, UK) for 3 ​h. TREK-MNPs were combined with Stro-1+ cells (25 μg/2 ​× ​10^5^ cells) and incubated for 1.5 ​h. TREK-MNP-labelled cells (TREK cells) were briefly washed in calcium-free DMEM before loading within hydrogels.

### 2.4 Growth factor-loaded microparticles

As previously described,^35^^,^^36^ a water-in-oil-in-water (w/o/w) emulsion method was employed to prepare poly (dl-lactide-co-glycolide) (P_DL_LGA, Lakeshore Biomaterials Inc., USA) microparticles. Selected growth factors were dissolved in 100 ​μL of 10 % (w/v) aqueous HSA (120 ​μg human recombinant VEGF [PeproTech, UK], 40 ​μg human recombinant TGF-β_3_ [PeproTech, UK], 0.8 ​mg PTHrP [PeproTech, UK], or 1 ​mg of human recombinant BMP-2 [‘in-house’ Hybridoma construct] and added to a solution of P_DL_LGA and P_DL_LGA-poly (ethylene glycol) (PEG)-P_DL_LGA triblock in dichloromethane to form a primary water-in-oil emulsion via homogenisation at 9000 ​rpm for 2 ​min using a Silverson L5M homogeniser (Silverson Machines, UK). Microparticles containing VitD_3_ were fabricated using a single oil-in-water emulsion method (25 ​μg of VitD_3_ in polymer solution (P_DL_LGA with 10 % P_DL_LGA-PEG-P_DL_LGA Triblock in dichloromethane) and homogenisation at 9000 ​rpm for 2 ​min). A double emulsion was then formed via homogenisation in 200 ​mL 0.3 % (w/v) poly (vinyl alcohol) (PVA) solution for 2 ​min at 9000 ​rpm (HSA/BMP-2) or 2000 ​rpm (HSA/VEGF and HSA/TGF-β_3_). Double emulsions were magnetically stirred at 300 ​rpm for a minimum 4 ​h before filtration and lyophilisation of the resultant microparticles. Large microparticles (50–100 ​μm) formulated with P_DL_LGA (85:15, 50 ​kDa) and 30 % (w/w) triblock copolymer, exhibited a fast release profile (HSA/VEGF, HSA/TGF-β_3,_ HSA/PTHrP), while small microparticles (20–30 ​μm) formulated with P_DL_LGA (50:50, 55 ​kDa) and 10 % (w/w) triblock copolymer, exhibited a slow release profile (HSA/BMP-2, HSA/VitD_3_). HSA alone was used as a control protein.

### 2.5 Alginate and bone ECM preparation

Decellularised and demineralised bone ECM digest (10 ​mg/mL) was prepared as previously described.^31^ Briefly, bovine cancellous bone was fragmented following freezing in liquid nitrogen and demineralised in 0.5 ​M HCl (25 ​mL/g bone) for 24 ​h at room temperature. Lipids were then removed with a chloroform/methanol solution (Fisher Scientific, UK) prior to washing with dH_2_O, snap freezing and lyophilisation. Demineralised bone was subsequently decellularised through agitation in 0.05 % trypsin/0.02 % EDTA at 37 ​°C for 24 ​h. The resultant bone ECM powder was stirred at room temperature for 96 ​h with 1 ​mg/mL pepsin in 0.01 ​M HCl until digested (final concentration of 10 ​mg/mL), aliquoted and stored at −20 ​°C. Low viscosity alginate (Acros Organics, Fisher Scientific, UK) was prepared as a 2 % (w/v) solution in calcium-free DMEM and pasteurised for 1 ​h at 65 ​°C. Control hydrogels included, i) bone ECM replaced with collagen (ALG/Col), and ii) overnight ultraviolet (U·V.) irradiation of bone ECM (irradiated ALG/ECM).^34^ These controls were designed to investigate the impact of retained bioactive components within bone ECM by comparing ALG/ECM with ALG/Collagen (ALG/Col) and inactivated (irradiated) bone ECM (irradiated ALG/ECM).

### 2.6 Hydrogel preparation and *in vivo* implantation

Human Stro-1+ cells (with and without TREK-MNP labelling) and growth factor-loaded microparticles were prepared as suspensions in calcium-free DMEM (1 ​× ​10^6^ cells/mL and 1 ​mg/μL, respectively). Dual growth factor combinations included HSA/VEGF/TGF-β_3_, HSA/VEGF/BMP-2, HSA/TGF-β_3_/BMP-2 (growth factor group one), or HSA/VEGF/PTHrP, HSA/VEGF/VitD_3_, HSA/TGF β3/PTHrP, HSA/TGF-β3/VitD_3_ (growth factor group two) were incorporated within the 2 % low-viscosity alginate component (5 ​μL microparticle suspension per growth factor). Final growth factor concentrations were 50 ​ng/mL (VEGF), 15 ​ng/mL (TGF-β_3_), 100 ​ng/mL (BMP-2), 100 ​ng/mL (PTHrP), and 25 ​nM (VitD_3_). Microparticle-loaded alginate (0.4 ​mL) was then combined with Stro-1+ cell suspensions (0.5 ​mL) and vortexed briefly for homogenisation. Microparticle- and cell-loaded alginate (0.9 ​mL) was then combined with bone ECM (0.6 ​mL) in a 3:2 ratio, and mixed thoroughly between two syringes attached by a luer lock (NHS Supplies, UK). Cylindrical hydrogel structures were prepared by pastette extrusion into 135 ​mM calcium chloride solution and incubation at room temperature for 10 ​min to allow cross-linking. Once set, hydrogels were incubated overnight in 2.7 ​mM calcium chloride-supplemented culture media (α-MEM, penicillin [100 U/mL], streptomycin [0.1 ​mg/mL] and l-ascorbic acid 2-phosphate [100 ​μM] at 37 ​°C in 5 % CO_2_ and a humidified atmosphere. Cylindrical hydrogels were cut into 5 ​mm length segments in preparation for subcutaneous implantation *in vivo* within MF1 nu/nu immuno-deficient mice bilaterally along the back (3 implants per side with a maximum of 6 implants per mouse; n ​= ​3 to 6 per group). One side received hydrogel implants with Stro-1+ cell incorporation, while the other side received hydrogel implants without Stro-1+ cell incorporation. Each mouse received one growth factor loaded hydrogel group. HSA/VEGF/TGF-β_3_ with TREK cells was repeated (n ​= ​6, in total) due to interesting results without BMP-2 incorporation. Implants were harvested after 28 days and fixed in 4 % paraformaldehyde (PFA). Additional hydrogels were prepared without Stro-1 enriched cell incorporation. Mechanotransduction was performed for 1 ​h every weekday over the 28-day implantation period, by placing mice with implanted TREK cells into a magnetic chamber. The chamber has static magnetics on either side providing a force of up to 100 ​pN per particle, between which the mice were allowed to move freely. Growth factors and loading concentrations were selected based on their previously investigated capacity to induce angio-, chondro- and osteo-genesis within SSC populations *in vitro (*unpublished data*).* Implants were harvested after 28 days and fixed in 4 % (w/v) paraformaldehyde (PFA). Additional hydrogels were prepared without Stro-1+ cell incorporation.

### 2.7 Micro-computed tomography

Harvested and fixed hydrogel samples were assessed by quantitative 3D analysis using a SkyScan 1176 scanning system (Bruker micro-CT, Kontich) and scanned at 18 ​μm resolution. NRecon software interface (v.1.6.4.6, Bruker micro-CT, Kontich) was used to reconstruct scanning data prior to analysis of bone volume (BV), tissue volume (TV), percentage bone volume (PBV), bone surface/volume ratio (BS/BV), trabecular number (Tb·N), trabecular thickness (Tb.T), and trabecular separation (Tb·S) using CT Analyser (v.1.13.2.1+, Bruker micro-CT, Kontich). Bone tissue was assessed using greyscale values 80–255, as wider values (60–255) began to highlight soft tissue. Soft tissue was assessed using greyscale values 20–255. Bone and soft tissue greyscale values were assessed according to standard phantom and intact mouse bone (forearm) scans.^34^

### 2.8 Histology

Macroscopic differences between hydrogels were visually assessed by stereomicroscopy (Leica, UK) using an attached PowerShot G10 camera (Canon) (Supplementary Fig. 1). Hydrogels were subsequently dehydrated through a series of ethanol washes and incubated in Histo-Clear (National Diagnostics, UK). Dehydrated samples were then incubated in molten paraffin wax for 1 ​h at 60 ​°C before embedding on an automated Shandon Citadel 2000. Two representative 7 ​μm thick sections across the diameter of each hydrogel (n ​= ​3 to 6 samples) were rehydrated through Histo-Clear, graded ethanol, and dH_2_O washes. Sections were then stained with Alcian blue/Sirius red (A/S), Von Kossa (VK), or Goldner's Trichrome (GT). A/S involved staining with Weigert's haematoxylin, 0.5 % Alcian blue, and 1 % Sirius red. VK involved staining with silver nitrate (U.V. irradiation), sodium thiosulfate, Alcian blue, and then van Gieson. GT involved staining with Weigert's haematoxylin, ponceau-fuchsin-azophloxin, phosphomolybdic acid, and then light green. Following dehydration (A/S and VK only), stained sections were mounted with DPX before imaging on an Olympus BX-51/22 dotSlide digital virtual microscope using OlyVIA 2.1 software (Olympus Soft Imaging Solutions, GmBH, UK).

### 2.9 Digital histology quantification

Hydrogel sections stained with A/S or VK were digitally analysed to quantify colour features as described previously.^34^^,^^42^ Colour threshold analysis of scanned images, using an optimised macro in Fiji (Image J),^43^ was performed to quantify growth parameters including tissue invasion, matrix deposition, mineralisation, and vascularisation. The contour of each hydrogel sample within scanned images was defined as the ‘region of interest’ (ROI) and additional sub-ROIs were created highlighting missing or torn areas of hydrogel; both were saved as an individual ROI corresponding to a single image. In brief, the macro functioned by thresholding a colour of interest (hue, saturation and brightness values; 0–255) for each histological stain. Hue, saturation and brightness values are provided in the footnotes of each histology figure. Thresholds were used to create a black (selected colour) and white (non-selected colour) mask. A ‘point grid’ was overlaid on the masks where each point constituted 5 by 5 pixels (approximate resolution of a single cell) and was set 50 pixels apart. ROIs were then applied to define areas for quantification within the mask images. Positive points (≥50 % black pixels) and total number of points within ROIs were quantified and used to calculate the colour of interest as a percentage of the total sample. Points quantified within sub-ROIs were evaluated where appropriate.

A/S stain colour quantification - blue (hue 120–150, saturation 50–255, brightness 0–255) represented proteoglycans and residual hydrogel, red (hue 210–255, saturation 20–255, brightness 0–255) represented tissue invasion, and purple (hue 140–200, saturation 50–255, brightness 140–255) represented collagen deposition.

VK stain colour quantification - black (hue 0–255, saturation 0–255, brightness 0–100) represented mineralisation, and pink (hue 170–255, saturation 100–255, brightness 50–255) represented cell invasion.

GT stain colour quantification - green represented collagen/osteoid, purple represented cell invasion, and red represented erythrocytes. Erythrocytes were representative of hydrogel vascularisation. A square grid (200 ​μm) was overlaid on each image and those squares containing bright red erythrocytes were counted and calculated as a percentage of the total number of squares covering the hydrogel sample. Erythrocyte quantification was not performed using the automated macro due to low occurrence and low surface area. The authors appreciate that GT is not a selective stain for erythrocytes, but found that bright red staining correlated with morphologically identified erythrocytes.

All hydrogel implants exhibited areas of dense stain which were torn and shredded. These areas were quantified and added to the total area of the immediate surrounding colour (blue in A/S stains, and black in VK stains).

### 2.10 Statistical analysis

Data presented as mean ​± ​standard deviation. Statistical analysis was performed using GraphPad InStat3 v3.06 software. Statistical significance between and within experimental groups was determined by a one-way ANOVA with Tukeys post-hoc test. Correlations between datasets were assessed using a linear regression model (Pearson). ‡ denotes positive correlation. Emboldened columns denote intra-group significance. Significance is depicted as ∗ P ​≤ ​0.05, ∗∗P ​≤ ​0.01, ∗∗∗P ​≤ ​0.001.

## 3 Results

### 3.1 Micro CT analysis of hydrogel mineralisation and bone formation

**Fig. 1:**
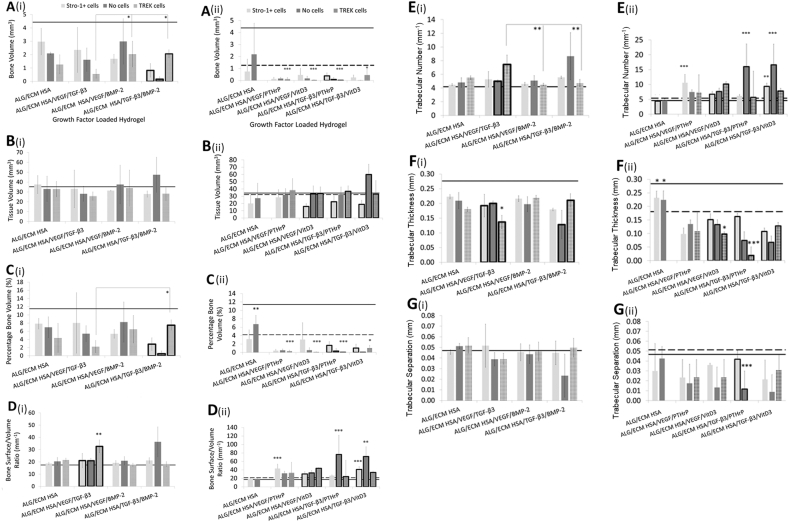
Micro CT analysis of hydrogels following 28 days *in vivo* implantation. Bone volume (**A**), tissue volume (**B**), percentage bone volume (**C**), bone surface to volume ratio (**D**), trabecular numbers (**E**), trabecular thickness (**F**) and trabecular separation (**G**) were all assessed between growth/osteoinductive factor groups. Emboldened columns depict statistically significant intra-group differences. The solid line marks the mean value recorded within control ALG/ECM. The dashed line marks the mean value recorded within control ALG/ECM HSA with TREK cells. Data represent mean ​± ​SD (N ​= ​3 hydrogels per group). ∗P ​≤ ​0.05, ∗∗P ​≤ ​0.01. ALG/ECM, alginate and bone extracellular matrix; HSA, human serum albumin; VEGF, vascular endothelial growth factor; TGF-β_3_, transforming growth factor beta 3; BMP-2, bone morphogenetic protein 2; PTHrP, parathyroid hormone-related protein; VitD3, vitamin D3; SD, standard deviation.

Following 28 days subcutaneous implantation, hydrogel constructs were recovered and assessed by micro CT for bone formation (Fig. 1A–G). Robust bone formation was observed in the presence of ALG/ECM and no groups in growth factor group one (Fig. 1A(i)-G(i)) were significantly different compared to the control group (depicted as a solid line) for all bone parameters assessed by micro CT analysis with the exception of the HSA/TGF-β_3_/BMP-2 group, which significantly reduced several bone parameters in the absence of Stro1+ cells. No significant differences were observed between the groups with Stro-1+ cell incorporation across all parameters investigated (Supplementary Fig. 3). Mechanotransduction within the HSA/VEGF/TGFβ_3_ group significantly (P ​≤ ​0.05) reduced several bone parameters (BV, PBV and Tb.T) compared to the other groups (Fig. 1A(i), 1C(i), and 1F(i), and Supplementary Fig. 4). This reduction correlated with an increase in BS/BV and Tb·N (Fig. 1D(i) and 1E(i)). Interestingly, intra-group analysis revealed that mechanotransduction reversed the observed decrease in BV, PBV and Tb.T within the HSA/TGFβ_3_/BMP2 group (Supplementary Fig. 5).

In contrast to growth factor group one, in the second osteoinductive/growth factor group most groups exhibited significantly (P ​≤ ​0.05) reduced bone formation parameters (BV, PBV and Tb.T) in comparison to control ALG/ECM (Fig. 1A(ii)-G(ii)). While intra-group analysis within the HSA/TGFβ_3_/PTHrP group demonstrated increased BV, PBV, Tb.T, and Tb·S with Stro-1+ cell incorporation only compared to analogous groups (and in the absence of mechanotransduction) (Supplementary Fig. 5). Mechanotransduction resulted in significantly (P ​≤ ​0.01) reduced Tb.T in comparison (Fig. 1F(ii)), and significantly (P ​≤ ​0.05) lower Tb·S was observed in comparison to both HSA only and HSA/TGF-β_3_/VitD_3_ groups undergoing mechanotransduction (Fig. 1G(ii)). No other differences across all data sets were observed in Tb·S.

The trend for reduced BV across most groups examined, correlated with a general increase in BS/BV. When Stro-1+ cells were incorporated, application of HSA/VEGF/PTHrP and HSA/TGF-β_3_/VitD_3_ groups demonstrated significantly (P ​≤ ​0.001) higher BS/BV compared to controls ALG/ECM and HSA (Fig. 1D(ii) and Supplementary Fig. 3). This also correlated with a significant increase in Tb·N (P ​≤ ​0.01) compared to the controls (Fig. 1E(ii)). These changes were reflected in the intra-group differences in the HSA/TGF-β_3_/VitD_3_ group. Mechanotransduction within the HSA/VEGF/VitD_3_ group also resulted in significantly (P ​≤ ​0.01) increased BS/BV and Tb·N, (Fig. 1**.**D(ii)-E(ii)). In the absence of Stro-1+ cell incorporation, both TGF-β_3_ containing groups exhibited significantly (P ​≤ ​0.05) higher BS/BV and Tb·N in comparison to controls (Fig. 1D(ii)-E(ii), and (Supplementary Fig. 4). However, following mechanotransduction, differences in BS/BV and Tb·N were no longer observed in these groups (Supplementary Fig. 6).

Soft tissue volume (TV) analysis showed no significant differences between growth factor/osteoinductive groups and control ALG/ECM (Fig. 1.B(ii)). However, intra-group analysis showed that incorporation of VitD_3_ or TGF-β_3_ groups with Stro-1+ cells resulted in significantly (P ​≤ ​0.05) lower TV (Fig. 1B(ii) and Supplementary Fig. 5). Interestingly, the negative effect on TV was reversed when incorporated Stro-1+ cells were under mechanotransduction.

### 3.2 Histological analysis of hydrogel mineralisation and bone formation

**Fig. 2:**
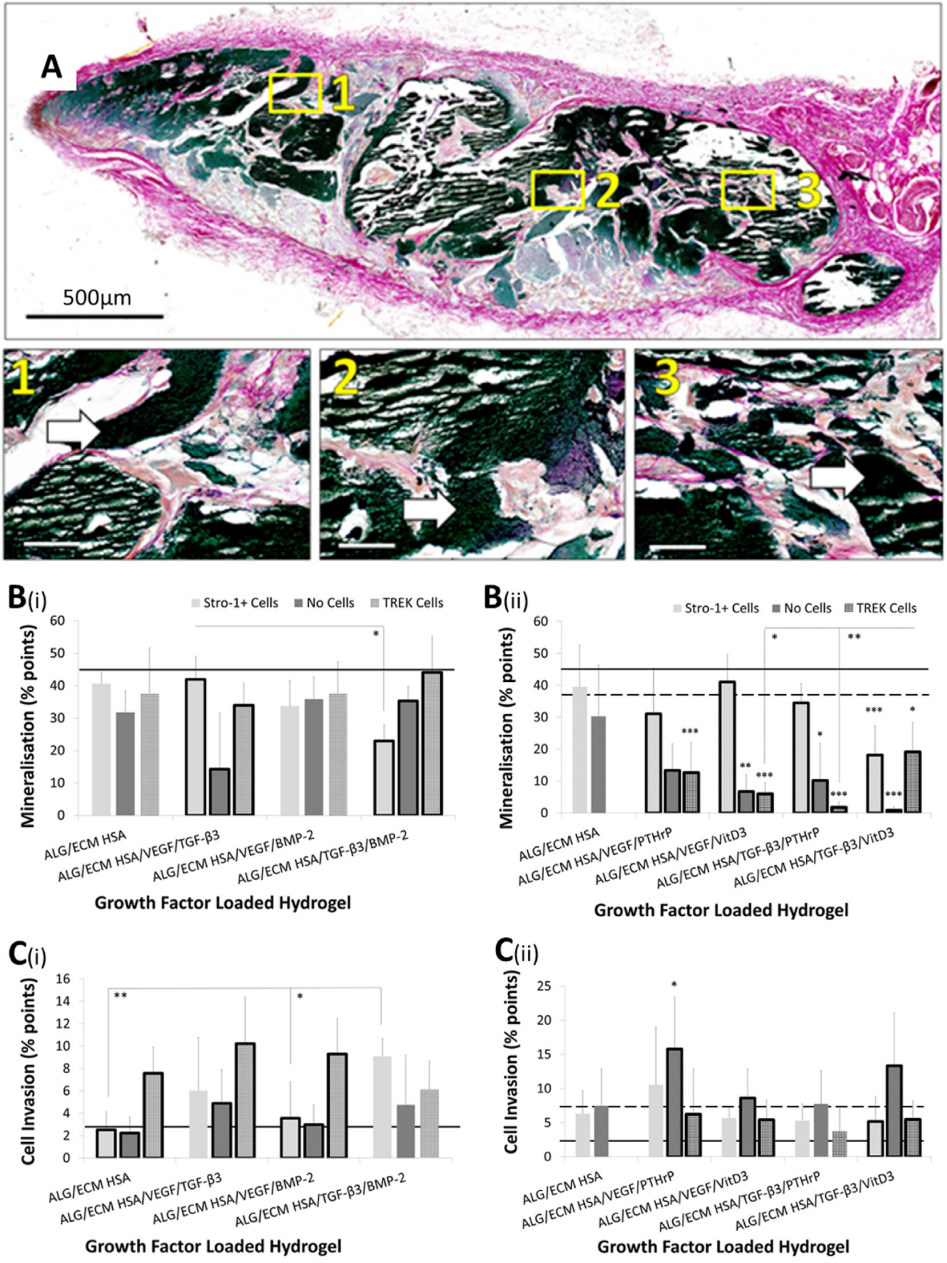
Hydrogel mineralisation between growth/osteoinductive factor groups following 28 days *in vivo* subcutaneous implantation. Samples were sectioned and stained with von Kossa (**A**). Gaps within tissues represent areas of torn and shredded tissue during sectioning (possible areas of high mineralization that crumbled during sectioning). Areas of dense mineralisation are depicted by white arrows within magnified areas. Representative image of a Von Kossa stained ALG/ECM HSA hydrogel following 28 days *in vivo* implantation is shown. Images were taken at low (single large images shown above – scale bar is 500 ​μm) and high magnifications (three smaller individual images shown above – scale bar is 50 ​μm). Colour quantification was through use of an optimised Image J macro.^34^ Black (hue 0–255, saturation 0–255, brightness 0–100) indicates mineralised tissue (**B**) and pink (hue 170–255, saturation 100–255, brightness 50–255) indicates cell invasion (**C**). Emboldened columns depict statistically significant intra-group differences. The solid line marks the mean value recorded within control ALG/ECM. Data represent mean ​± ​SD (N ​= ​3 hydrogels per group). ∗P ​≤ ​0.05, ∗∗P ​≤ ​0.01, ∗∗∗P ​≤ ​0.001. ALG/ECM, alginate and bone extracellular matrix; HSA, human serum albumin; VEGF, vascular endothelial growth factor; TGF-β_3_, transforming growth factor beta 3; BMP-2, bone morphogenetic protein 2; PTHrP, parathyroid hormone-related protein; VitD3, vitamin D3; SD, standard deviation.

**Fig. 3:**
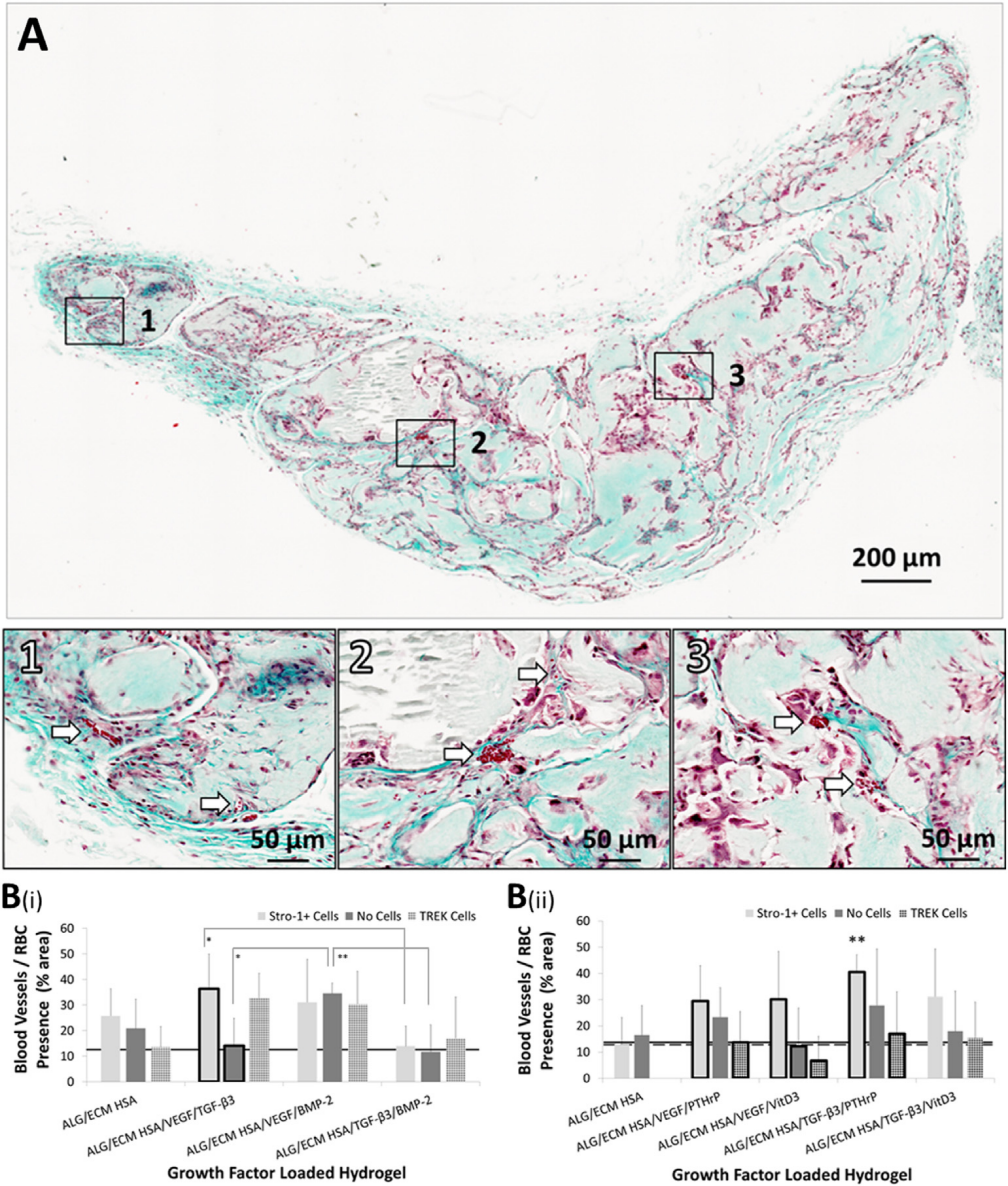
Histological analysis of hydrogels stained with Goldner's Trichrome. Hydrogels were subcutaneously implanted within immunodeficient mice for 28 days. Extensive vascularisation throughout the implanted hydrogel structure depicted by the presence of erythrocytes (**A**), a square grid (200 ​μm) overlay was used to quantify erythrocytes. Host blood vessel invasion is depicted by white arrows within magnified areas. Representative image was taken of a Goldner's Trichrome stained ALG/ECM HSA/TGF-β_3_/BMP-2 hydrogel following 28 days *in vivo* implantation. Comparison between all groups with Stro-1+ cells, without Stro-1+ cells or with TREK cells were assessed by a one-way ANOVA with Tukeys post-hoc test (**B**). Emboldened columns depict statistically significant intra-group differences. For comparisons between control groups refer to 34. The solid line marks the mean value recorded within control ALG/ECM. The dashed line marks the mean value recorded within control ALG/ECM HSA with TREK cells. NS indicates ‘no significance’. Data represent mean ​± ​SD (N ​= ​3 hydrogels per group). ∗P ​≤ ​0.05, ∗∗P ​≤ ​0.01, ∗∗∗P ​≤ ​0.001. ALG/ECM, alginate and bone extracellular matrix; HSA, human serum albumin; VEGF, vascular endothelial growth factor; TGF-β_3_, transforming growth factor beta 3; BMP-2, bone morphogenetic protein 2; PTHrP, parathyroid hormone-related protein; VitD3, vitamin D3; SD, standard deviation.

Macroscopic differences were visually assessed following micro-CT analysis using stereomicroscopy (for ALG/ECM, see 34) (Supplementary Fig. 1). No major differences in the appearance (shape, size, or texture) of the hydrogel constructs were observed between growth factor groups or within the groups. All hydrogel constructs displayed similar findings with large opaque white areas which were hard and rigid upon manipulation; indicative of mineralisation. Indeed, microscopic histological analysis clearly demonstrated the presence of dense black mineral deposits in VK stained hydrogel cross-sections which were uniformly torn and shredded following microtome sectioning; a typical issue when sectioning mineralised tissue (Fig. 2A). Further examination revealed host tissue invasion and vascularisation. Goldner's trichrome GT) stained sections highlighted the presence of erythrocytes and blood vessels throughout the hydrogel structure (Fig. 3A), with larger vessels visible to the naked eye (Supplementary Fig. 1). Although all groups investigated displayed some degree of mineralisation, tissue invasion, and vascularisation, closer analysis using Image J macro^34^ was performed to assess potential differences. The macro quantified colours representative of shared biological processes active within the hydrogel constructs, across each histological stain, enabled statistical assessment of possible differences in bone formation.

### 3.3 Alcian blue/sirius red – proteoglycan/collagen deposition

**Fig. 4:**
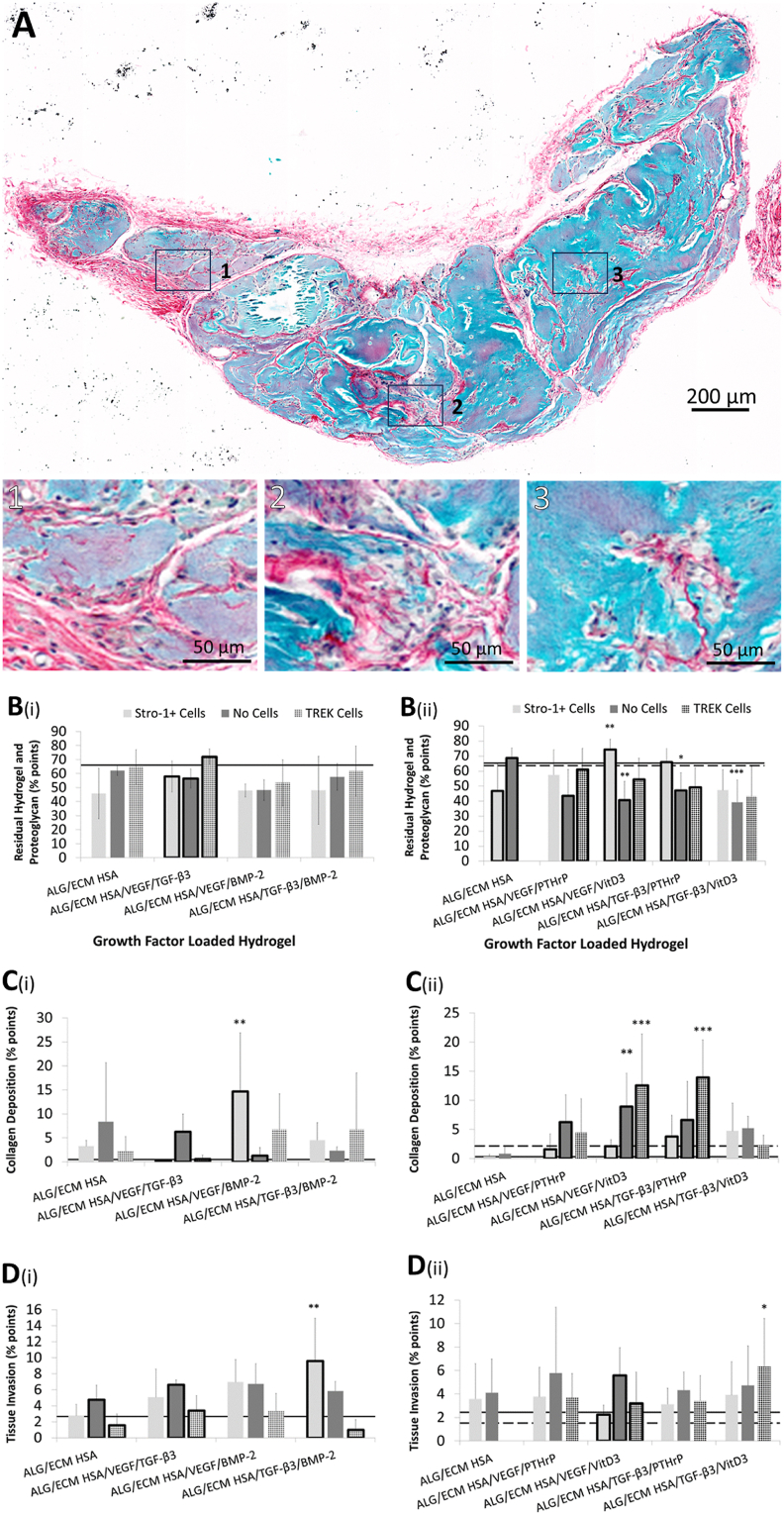
Histological analysis of hydrogels stained with Alcian blue/Sirius red. Hydrogels were subcutaneously implanted within immunodeficient mice for 28 days. Samples were sectioned and stained with Alician blue/Sirius Red. Representative image was taken of an Alcian blue/Sirius red stained ALG/ECM HSA/TGF-β_3_/BMP2 hydrogel following 28 days *in vivo* implantation (**A**). Colour quantification was through the use of an optimised Image J macro.^34^ Blue (hue 120–150, saturation 50–255, brightness 0–255) indicated residual hydrogel and proteoglycan deposition (**B**), purple (hue 140–200, saturation 50–255, brightness 140–255) indicated collagen deposition within the hydrogel (**C**) and red (hue 210–255, saturation 20–255, brightness 0–255) indicated tissue invasion (**D**). Emboldened columns depict statistically significant intra-group differences. The solid line marks the mean value recorded within control ALG/ECM. The dashed line marks the mean value recorded within control ALG/ECM HSA with TREK cells. Data represent mean ​± ​SD (N ​= ​3 hydrogels per group). ∗P ​≤ ​0.05, ∗∗P ​≤ ​0.01, ∗∗∗P ​≤ ​0.001. ALG/ECM, alginate and bone extracellular matrix; HSA, human serum albumin; VEGF, vascular endothelial growth factor; TGF-β_3_, transforming growth factor beta 3; BMP-2, bone morphogenetic protein 2; PTHrP, parathyroid hormone-related protein; VitD3, vitamin D3; SD, standard deviation.

**Fig. 5:**
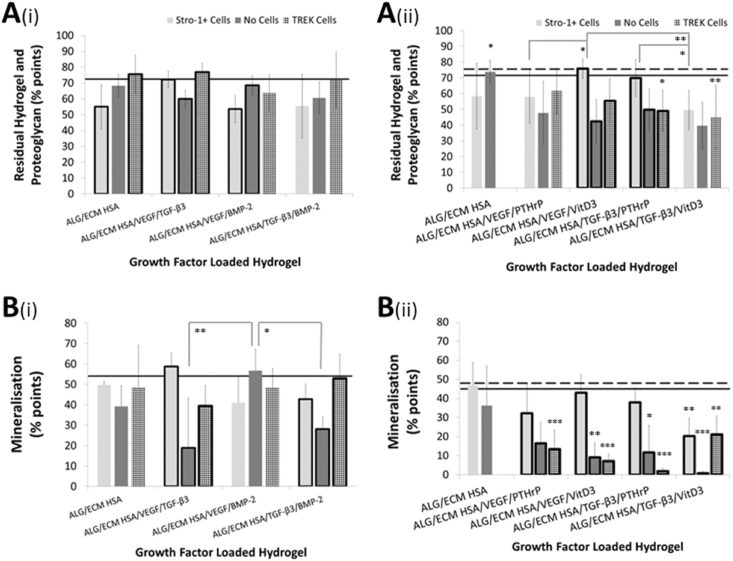
Histological analysis of torn/shredded areas within hydrogel samples following Alcian blue/Sirius red (**A**) and Von Kossa stain (**B**). Hydrogels were subcutaneously implanted within immunodeficient mice for 28 days. Colour quantification was through the use of an optimised Image J macro.^34^ Blue indicated residual hydrogel and proteoglycan deposition (**A**) and black indicated mineralisation (**B**). Comparison between all groups with Stro-1+ cells, without Stro-1+ cells, or with TREK cells were assessed by a one-way ANOVA with Tukeys post-hoc test. Emboldened columns depict statistically significant intra-group differences. The solid line marks the mean value recorded within control ALG/ECM. The dashed line marks the mean value recorded within control ALG/ECM HSA with TREK cells. NS indicates ‘no significance’. Data represent mean ​± ​SD (N ​= ​3 hydrogels per group). ∗P ​≤ ​0.05, ∗∗P ​≤ ​0.01, ∗∗∗P ​≤ ​0.001. ALG/ECM, alginate and bone extracellular matrix; HSA, human serum albumin; VEGF, vascular endothelial growth factor; TGF-β_3_, transforming growth factor beta 3; BMP-2, bone morphogenetic protein 2; PTHrP, parathyroid hormone-related protein; VitD3, vitamin D3; SD, standard deviation.

Control non-implanted hydrogels labelled strongly for Alcian blue possibly due to retained proteoglycan within the bone ECM component. Therefore, residual implanted hydrogel and new proteoglycan deposition were indistinguishable from one another (Fig. 4A).^34^ In growth factor group one no significant differences were observed between all groups investigated (Fig. 4B(i)) and (Supplementary Fig. 6). Furthermore, intra-group analysis revealed minimal differences. In the absence of mechanotransduction, residual hydrogel and proteoglycan deposition was significantly (P ​≤ ​0.05) reduced within the HSA/VEGF/TGF-β_3_ group. Similar results were observed even when torn and shredded areas on sections were included in the analysis (considered to be the same colour as the surrounding tissue), indicating minimal impact on accurate readouts (Fig. 5A(i)-B(i)) and Supplementary Fig. 7).

In growth factor group two Stro-1+ cell incorporated groups with VEGF/VitD_3_ (not under mechanotransduction) exhibited significantly (P ​≤ ​0.01) increased residual hydrogel and proteoglycan deposition compared to the HSA control group (Fig. 4B(ii) and Supplementary Fig. 7). Without Stro-1+ cell incorporation, no inter-group differences were observed except for control HSA exhibiting significantly (P ​≤ ​0.05) higher residual hydrogel and proteoglycan deposition compared to most other groups (besides VEGF/PTHrP). Intra-group analysis broadly reflected the differences observed between growth factor/osteoinductive groups (Supplementary Fig. 8).

Collagen deposition was assessed and quantified within Alcian blue/Sirius red stained sections. Collagenous matrix typically appears red, however due to substantial Alcian blue background labelling of the hydrogels, new collagen deposition appeared purple in all sections. On initial examination, a number of groups within growth factor group one exhibited enhanced collagen deposition compared to control hydrogels (Fig. 4C(i)). However, only HSA without Stro-1+ cell incorporation and HSA/VEGF/BMP-2 with Stro-1+ cell incorporation exhibited significantly (P ​≤ ​0.05) increased collagen deposition compared with control hydrogels (Supplementary Fig. 6). Interestingly, mechanotransduction within the HSA/VEGF/TGF-β_3_ group resulted in a small but statistically significant (P ​≤ ​0.05) increase in collagen deposition compared with control ALG/ECM (Supplementary Fig. 6). However, when compared to acellular gels, Stro-1+ cell incorporation significantly (P ​≤ ​0.001) limited collagen deposition within the HSA/VEGF/TGF-β_3_ group (Supplementary Fig. 8). The reverse was observed within the HSA/VEGF/BMP-2 group, where the presence of Stro-1+ cells increased collagen deposition.

In growth factor group two, TGF-β3 containing groups with Stro-1+ cells significantly (P ​≤ ​0.05) increased collagen deposition compared to ALG/ECM only, while in the absence of cells, the VEGF groups and the TGF-β3/PTHrP group also increased collagen deposition (Fig. 4C and Supplementary Fig. 7). Following mechanotransduction, both HSA/VEGF/VitD3 and HSA/TGF-β3/PTHrP exhibited significantly (P ​≤ ​0.001) increased collagen deposition compared to all other groups. Interestingly, it appears Stro-1+ cell incorporation within HSA/VEGF/VitD3 had a significant (P ​≤ ​0.05) negative effect on collagen deposition, which mechanical stimulation was able to rescue (Supplementary Fig. 8).

Host tissue invasion, shown as fibrous channels spanning the hydrogel diameter were quantified. In growth factor group one, constructs with BMP-2 and Stro-1+ cell incorporation exhibited significantly (P ​≤ ​0.05) increased tissue invasion compared to control group ALG/ECM (Fig. 4D(i) and Supplementary Fig. 6). Without Stro-1+ cell incorporation, all growth factor groups demonstrated significantly (P ​≤ ​0.05) increased tissue invasion compared to ALG/ECM controls. Mechanotransduction appeared to reduce tissue invasion with no significant differences observed between groups (Supplementary Fig. 6). Intra-group analysis supported significant (P ​≤ ​0.05) reduction in tissue invasion amongst those with mechanotransduction compared to those without (Supplementary Fig. 8). In growth factor group two, no significant differences between growth factor/osteoinductive groups were observed independent of Stro-1+ cell incorporation (Fig. 4D(ii) and Supplementary Fig. 6). Only HSA/TGFβ_3_/VitD_3_ exhibited significantly (P ​≤ ​0.05) increased tissue invasion compared to controls ALG/ECM and HSA following mechanotransduction. Intra-group analysis revealed a significant (P ​≤ ​0.05) decrease in tissue invasion within the HSA/VEGF/VitD_3_ group upon Stro-1+ cell incorporation with and without mechanotransduction (Supplementary Fig. 8).

Regarding the breakdown of sample colouration, encouragingly, where residual hydrogel and proteoglycan quantification accounted for up to 70 % of the sample, and collagen quantification accounted for up to 15 %, host tissue invasion quantification accounted for ≤10 %. Together, colour quantification accounted for approx. 95 % of the hydrogel sample area with torn and shredded areas accounted for the remaining 5 % (Fig. 5A and Supplementary Fig. 7).

### 3.4 Von Kossa – tissue invasion/mineralisation

All groups investigated demonstrated areas of mineralised tissue (Fig. 2). Closer analysis revealed that growth factor groups with BMP-2 and Stro-1+ cell incorporation exhibited significantly (P ​≤ ​0.05) lower mineralisation compared with control ALG/ECM. Whilst within the VEGF/TGF-β3 group (with Stro-1+ cells) mineralisation was similar to control ALG/ECM (Fig. 2B(i)). In the absence of Stro-1+ cell incorporation the relative reduction in mineralisation within BMP-2 containing groups was not observed (Fig. 2B(i), Supplementary Fig. 9). While the HSA/VEGF/TGF-β_3_ group (without cells) showed a significant (P ​≤ ​0.001) reduction in mineralised tissue compared with control ALG/ECM. Mechanotransduction within this group rescued mineralisation to some degree, but not to the same extent as having Stro-1+ cells alone. In contrast, mechanotransduction within the TGF-β3/BMP-2 group rescued mineralisation to similar levels as the ALG/ECM control. Similar results were observed even when torn and shredded areas were included (Fig. 5B(i) and Supplementary Fig. 7). In growth factor group two, no group exhibited greater mineralisation compared to controls ALG/ECM and HSA. Indeed all but one group, HSA/VEGF/VitD_3_ with Stro-1+ cell incorporation, exhibited significantly (P ​≤ ​0.01) lower mineralisation in comparison to control ALG/ECM (Fig. 2B(ii) and Supplementary Fig. 9). Stro-1+ cell incorporation appeared to have a positive effect on mineralisation within all growth factor/osteoinductive groups showing significant (P ​≤ ​0.01) intra-group variation (Supplementary Fig. 8). Mechanotransduction reversed this positive effect within all groups except HSA/TGF β3/VitD_3_. Following mechanotransduction, this group also demonstrated significantly (P ​≤ ​0.05) increased mineralisation compared to HSA/VEGF/VitD_3_ and HSA/TGF β3/PTHrP (Supplementary Fig. 9).

Cell cytoplasm and nuclei were quantified to assess host cell invasion (Fig. 2). Cell invasion within the HSA/TGF-β_3_/BMP-2 with Stro-1+ cells group was significantly (P ​≤ ​0.05) greater than most other groups and controls HSA and ALG/ECM (Fig. 2C(i)). No significant differences were observed between groups without Stro-1+ cell incorporation (Supplementary Fig. 9). In addition, mechanotransduction within VEGF incorporated groups demonstrated equivalent significantly (P ​≤ ​0.01) increased cell invasion compared to controls ALG/ECM. Intra-group analysis within these same groups also demonstrated significantly (P ​≤ ​0.05) increased cell invasion following mechanotransduction (Supplementary Fig. 8). In growth factor group two, HSA/VEGF/PTHrP with Stro-1+ cell incorporation exhibited significantly (P ​≤ ​0.05) increased host cell invasion compared to ALG/ECM and was increased further in the absence of cells. HSA/TGF β3/VitD_3_ without Stro-1+ cells also significantly increased cell invasion compared to ALG/ECM control (Fig. 2C(ii) and Supplementary Fig. 9). Mechanotransduction had a similar effect to Stro-1+ cells alone on cell invasion (Supplementary Fig. 8).

### 3.5 Goldner's trichrome – cell invasion/vascularisation

All groups exhibited a degree of vascular invasion with host blood vessels visible in most cross-sections following histological analysis (Fig. 3). In growth factor group one HSA/VEGF/BMP-2 without Stro-1+ cell incorporation exhibited significantly (P ​≤ ​0.01) greater blood vessel invasion compared to control ALG/ECM (Fig. 3B(i) and Supplementary Fig. 10). In contrast, only with Stro-1+ cell incorporation did the HSA/VEGF/TGF-β_3_ group exhibit significantly (P ​≤ ​0.05) increased host blood vessel invasion compared to control ALG/ECM. Mechanotransduction had a similar, but not significant, effect as Stro-1+ cells alone across all growth factor groups. This was reflected by the lack of significant intra-group difference in comparison to those without mechanotransduction (Supplementary Fig. 8). No further significant intra-group differences were observed within groups in growth factor group one. In growth factor group two statistical analysis revealed a significant (P ​≤ ​0.01) increase in vascularisation between HSA/TGF β3/PTHrP with Stro-1+ cell incorporation and both controls ALG/ECM and HSA (Fig. 3B(ii) and Supplementary Fig. 10). Intra-group differences revealed that mechanotransduction reversed the increased vascularisation following Stro-1+ cell addition across most groups (Supplementary Fig. 8).

### 3.6 Correlations between digital histology parameters

Further analysis investigated correlations between histological stains, thereby interrogating internal mechanisms of host invasion and cellular colonisation. Overall, similar patterns were observed between groups, independent of Stro-1+ cell incorporation and mechanotransduction across both data sets. Residual hydrogel and proteoglycan deposition negatively correlated (P ​≤ ​0.05) with both collagen deposition and tissue invasion, while these two positively correlated (P ​≤ ​0.05) with each other (Supplementary Fig. 11, Supplementary Fig. 12). Interestingly, and perhaps intuitively, cell invasion positively correlated (P ​≤ ​0.05) with tissue invasion, independent of Stro-1+ cell incorporation and mechanotransduction. Differences were observed however, dependent on the presence of Stro-1+ cells, including a positive correlation (P ​≤ ​0.01) between vascularisation and both collagen deposition and mineralisation, which was comparable across both data sets. However, only in groups without Stro-1+ cell incorporation were negative correlations observed between residual hydrogel and vascularisation (P ​≤ ​0.05), and between mineralisation and collagen deposition/tissue invasion (P ​≤ ​0.05), which was comparable across both datasets. Most correlations observed between histological parameters following mechanotransduction, were shared with those without mechanical stimulation (Supplementary Fig. 12). The only correlation not shared with groups without mechanotransduction was a positive correlation (P ​≤ ​0.05) between cell invasion and vascularisation observed within growth factor group one. Mineralisation did not correlate with any other stain in groups containing Stro-1+ cells within growth factor group two. However, following mechanotransduction, a positive correlation (P ​≤ ​0.05) with residual hydrogel and proteoglycan deposition, and negative correlation (P ​≤ ​0.001) with collagen deposition was observed. These correlations were shared in groups without Stro-1+ cell incorporation. Further correlations were largely observed between stains from groups without Stro-1+ cell incorporation. Here, cell invasion positively correlated (P ​≤ ​0.001) with collagen deposition, negatively correlated (P ​≤ ​0.001) with both residual hydrogel and proteoglycan deposition and mineralisation. Vascularisation positively correlated (P ​≤ ​0.05) with collagen deposition and cell invasion.

## 4 Discussion

The current study has investigated *in vivo* bone formation within hybrid growth factor or osteoinductive factor-loaded hybrid ALG/ECM hydrogels seeded with cells and exposed to mechanotransduction through MNP targeted to the TREK1 ion channel, as candidate implants for skeletal repair. We have previously shown that control ALG/ECM hydrogels exhibit extensive tissue mineralisation and bone formation.^34^ Bone formation in the current study was not found to be enhanced by the addition of SSCs (with and without mechanotransduction) or growth factor/osteotropic combinations (at the concentrations examined). This observation is in broad agreement with our previous study which examined single growth factor delivery *in vivo*. The results of that study also showed that release of growth factor did not enhance bone formation over ALG/ECM gels alone (and in the case of TGF-β3 reduced bone formation in gels). While BMP-2 and VitD3 also appeared to restore bone formation towards levels similar to the control in that study.^34^ Nonetheless, ALG/ECM serves as a useful and robust benchmark to measure the effect of controlled release of dual growth factors with and without SSCs and mechanotransduction. In the present study, the osteoinductive capacity of control ALG/ECM was most likely due to inherent growth factors within the bone ECM component retained following processing.^31^^,^^44^^,^^45^ This hypothesis is supported by reduced bone formation observed within irradiated ALG/ECM samples. Exposure to UV can cause growth factor and alginate degradation, but also increased cross-linking within the ECM component,^46^ all of which would negatively affect the bone formation capacity observed in ALG/ECM hydrogels.

Although incorporation of SSCs and growth factor combinations did not enhance the osteoinductive capacity of ALG/ECM, significant differences were observed in the process of bone formation and resultant structure. Indeed, BMP-2 activity was modulated by combination with VEGF or TGFβ_3_, leading to improved or reduced bone formation via downstream effects on host-derived hydrogel vascularisation. Growth factor combination alone demonstrated minimal impact on bone formation following micro-CT analysis. However, spatiotemporal release of chondrogenic followed by osteogenic growth factors, within groups without Stro-1+ cell incorporation, resulted in reduced bone formation, evidenced by thinner trabeculae, in comparison to control ALG/ECM. This may be due to the temporal release of TGFβ_3_ and BMP-2 in sequence, leading to rapid mineralisation before additional matrix deposition could take place, resulting in lower endpoint bone volume. Alternatively, this may be a consequence of a lack of VEGF-induced host-derived vascularisation, and thus SSC recruitment. Indeed, incorporation of Stro-1+ cells within this group restored bone volume, evidenced by thicker trabeculae, which was significantly enhanced with mechanical stimulation through TREK1. However, an interesting finding is that mechanotransduction appeared to act in concert only with BMP-2 induction. Previous studies have demonstrated a link between BMP-2 and mechanotransduction where mechanical forces can positively regulate and enhance SMAD signalling,^44^^,^^45^^,^^47^ which results in increased mineralisation and improved bone healing.^14^ The results in the current study correlate with our previous work which showed that combining BMP-2 induction alongside mechanotransduction through TREK1 can augment mineralisation in collagen gels *in vitro* and bone formation in an *ex vivo* chick femur model.^48^ Furthermore, mechanotransduction alone through TREK1 was also shown to enhance collagen and calcium deposition in co-cultures of chick epiphyseal cells and hMSC *in vitro*.^40^ In the present study, without direct osteogenic induction from BMP-2 incorporation, hydrogels with mechanotransduction exhibited considerably less bone formation. Analysis of bone-related parameters suggests that mechanical stimulus without osteogenic induction results in the formation of many thin trabeculae, rather than fewer thick trabeculae.

Tissue volume remained largely unaffected by osteotropic factor delivery from growth factor group two (incorporating PTHrP or VitD_3_). However, exposure of incorporated Stro-1+ cells to either TGFβ_3_ or VitD_3_ resulted in significantly reduced tissue volume, again potentially due to rapid terminal differentiation towards an osteogenic phenotype, reducing the opportunity to deposit new matrix prior to mineralisation. Indeed, possible disruption or re-direction of differentiation following mechanotransduction through TREK1 reversed this loss in tissue volume. Although PTHrP and VitD_3_ are osteotropic factors, their function can be modulated, consequently leading to structural changes in bone formation. Combination of VEGF with PTHrP and TGFβ_3_ with VitD_3_ led to the formation of numerous thin trabeculae, which in turn increased the bone surface to volume ratio compared to controls. Reversing the combinations, TGFβ_3_ with PTHrP and VEGF with VitD_3_, modulated their osteotropic activity with altered structural bone parameters observed. However, removal of Stro-1+ cells or addition of mechanotransduction, respectively within these groups, reinstated their original function and led to increased thin trabeculae formation with increased bone surface to volume ratio. These results highlight that cells require both the correct biochemical and physical cues, applied in the right sequence, to successfully form mature mineralised matrix.

The hydrogels were subsequently assessed histologically to investigate more closely the internal processes leading to bone formation, host tissue infiltration and new matrix deposition. Following implantation, hydrogels were slowly resorbed and replaced by new collagen deposition or host tissue invasion over 28 days. Host cells were observed colonising the implanted hydrogels, depositing collagen and forming new matrix as a fibrous network throughout the hydrogel structure which was independent of growth factor combination. These networks appeared to infiltrate around areas of alginate suggesting the bone ECM component presented invasion points and guided cell growth internally. Host cell invasion was also accompanied by host blood vessel ingrowth. It is possible therefore, that these hybrid constructs demonstrate propensity for improved integration within host tissues leading to enhanced defect repair, subject to effective vascularisation and induction of implanted Stro-1+ cells. Minimal differences in residual hydrogel and proteoglycan deposition between groups demonstrated similar breakdown and/or resorption following implantation. In fact, addition of Stro-1+ cells without osteotropic factor induction further enhanced hydrogel resorption/replacement in some groups. However, mechanotransduction without BMP-2 induction appeared to slow hydrogel resorption. Indeed, slow resorption accompanied lower host tissue invasion and less bone formation, observed by micro-CT analysis. In support, only Stro-1+ cell-incorporated hydrogels with BMP-2 induction demonstrated increased host tissue invasion compared to controls, yet these groups demonstrated reduced mineralisation. Without osteoinduction, Stro-1+ cell incorporation led to both reduced tissue invasion and collagen deposition. Conversely without the presence of SSCs, host tissue invasion was greater in all groups compared with controls, suggesting SSC addition alone restricted host infiltration. Simple addition of BMP-2 did not always reverse the negative effect of SSC addition however; combination with an angio- *or* chondrogenic growth factor appeared to modulate BMP-2 function. For example, only combination with VEGF reversed the negative effect of SSC addition on collagen deposition within the hydrogel structure.

Upon induction with either VEGF/VitD_3_ or TGF β3/PTHrP, new collagen deposition was reduced, possibly due to rapid osteogenic differentiation. In support, mechanotransduction through TREK1 within these same groups appeared to disrupt or re-direct differentiation towards a non-osteogenic (decreased mineralisation), possibly chondrogenic phenotype (increased collagen deposition). Interestingly, most groups demonstrated significantly lower mineralisation compared to control ALG/ECM and addition of Stro-1+ cells within VEGF and PTHrP groups merely maintained these levels of mineralisation.

Mechanical stimulation exhibited a small but significant decrease in host cell invasion compared to equivalent groups without Stro-1+ cell incorporation. This suggests that Stro-1+ cells, upon mechanical induction within the current system, differentiate towards a non-osteogenic lineage. Mechanotransduction-facilitated differentiation towards a chondrogenic phenotype would lead to formation of cartilaginous matrix, predominantly free of vasculature and potentially generating signals which inhibit host-derived vascularisation and therefore accompanying host cell invasion. VEGF indeed brought its own merits of increased host cell invasion compared to controls; an essential precursor to increased tissue invasion and collagen deposition. The action of VEGF appeared dependent on mechanical stimulation however, suggesting that implanted SSCs may have signalled host cell invasion following mechanotransduction, while VEGF then directed differentiation towards the angiogenic lineage. Indeed, without conflicting osteogenic induction, VEGF successfully induced greater vascularisation alongside SSC signalling for host cell invasion. Interesting to note here is that BMP-2 only conflicts with VEGF signalling when in the presence of incorporated Stro-1+ cells. Without Stro-1+ cells, HSA/VEGF/BMP-2 incorporation also led to greater vascularisation compared to controls. Vascularisation was reduced upon chondrogenic TGFβ_3_ signalling leading to cartilaginous tissue formation which is inherently and natively void of vasculature. However, SSC addition in combination with VEGF reversed this reduction in vascularisation. Combinations with BMP-2 did not improve vascularisation, but not through a lack of host infiltration because HSA/TGFβ_3_/BMP-2 signalling resulted in enhanced host cell invasion compared to controls. Vascularisation within growth factor group two was significantly reduced following the current regime of mechanical stimulation. The combination of osteogenic and chondrogenic induction interestingly reduced mineralisation within hydrogels in the presence of SSCs, as opposed to increased bone formation. In this scenario, osteochondral induction may have led to rapid mineralisation before formation of osteoid could take place; BMP-2 osteoinduction resulted in lower mineralisation compared to controls. In support, without SSC incorporation, mineralisation was unaffected; which may be due to the absence of inducible cells within the hydrogel to drive rapid mineralisation, and host tissue/cells had time to infiltrate the hydrogel structure prior to mineralisation. Interestingly, mechanotransduction within the HSA/TGF-β_3_/BMP-2 group reversed the low levels of mineralisation observed, potentially through disruption of osteoblast differentiation and/or function aiding additional osteoid formation prior to mineralisation.

Correlation analysis of internal processes within hydrogels revealed that hydrogel volume slowly reduced, replaced by new matrix formation derived from host cell infiltration and colonisation. As host cells and tissue permeated the hydrogel structure, new collagen matrix was deposited. Stro-1+ cells within the hydrogel enhanced blood vessel invasion alongside new collagen deposition, delivering additional host-derived SSCs which, upon appropriate osteoinduction, drove tissue mineralisation and bone formation. Without Stro-1+ cell incorporation, host invasion and collagen deposition remained unaffected, however new matrix formation exhibited reduced mineralisation, suggesting that the presence of Stro-1+ cells was important for bone formation and host cell recruitment in some groups. The potential implications are reduced host-derived osteoprogenitor cells for bone formation, and/or reduced osteogenic differentiation.

There are several limitations that should be considered when interpreting our findings. In this study we used alginate-based hydrogels as a carrier for SSCs and growth factor-loaded microparticles. Although alginate has been used within the clinical setting for several decades (e.g. wound dressings) further investigation would be required to ascertain whether the hydrogel system would elicit an immune response. The use of alginate also required the application of the cross-linking agent calcium chloride. It is important to note, Lee C. S. et al.^49^ reported that calcium chloride cross-linked, rather than barium chloride cross-linked, alginate microbeads implanted subcutaneously within mice display passive calcification. Furthermore, in other studies, the concentration of calcium ions *in vitro* has been shown to affect osteoblast mineralisation in a dose responsive manner.^50^^,^^51^ We also observed passive calcification of our subcutaneously implanted hybrid hydrogels, which may affect on our analysis. However, our analysis suggests that this does not significantly impact on the results reported in this study given: i) quantification of torn/shredded areas, suggestive areas of calcified alginate, did not significantly alter comparisons between groups and, ii) residual implanted hydrogel (blue quantification) did not correlate with areas of mineralisation (black quantification). Indeed, given all hydrogels contained similar levels of alginate, one could expect similar levels of passive calcification which would not explain the significant inter-group differences observed.

In the current study a single batch of ECM was used to construct the hydrogels. Use of bone ECM from different sources can introduce batch variability in the amount of residual growth factors after processing. In the future, batch variability in residual growth factor content could be accounted for by tailoring the quantities of additional growth factor-loaded microparticles. Although cells and microparticles were homogenised within the hydrogels, their distribution was not assessed, and thus this could have created unintended cell/osteoinductive factor gradients within the hydrogel constructs impacting on cell behaviour and host invasion. In addition, SSCs were not tracked post-implantation and therefore it is not possible to distinguish tissue formation originating from the implanted cells versus host tissue invasion.

Finally, the subcutaneous implant model is not typically conducive for creating ectopic bone because it is not subjected to the same mechanical stimulus as load-bearing bone. Mechanotransduction directed towards TREK1 channels, using MNPs and magnetic fields, were employed in the current study to address this lack of mechanical stimulus in the subcutaneous model. However, while these forces were applied directly to the cells, the forces involved may not directly compare with the forces observed in bone undergoing physiological load.

## 5 Conclusions

Hybrid ALG/ECM hydrogels containing SSCs and growth/osteotropic factor-releasing microparticles in combination with magnetic particle-mediated mechanotransduction were assessed for *in vivo* bone formation capacity. Control hydrogels exhibited extensive tissue mineralisation which neither SSC (with and without mechanotransduction through TREK1) nor growth factor incorporation further enhanced. This was most likely due to inherent growth factors within the bone ECM component following processing. However, SSC incorporation and growth factor combinations displayed significant effects on the process of tissue formation and resultant bone structure, which were further regulated and fine-tuned by TREK1-mediated mechanotransduction increasing collagen matrix deposition together with PTHrP and VitD_3_. Stro-1+ cell presence with PTHrP or VitD_3_ induction enhanced bone formation, whilst BMP-2 activity was modulated following combination with either VEGF or TGF-β_3_, either improving or reducing bone formation via downstream effects on host-derived hydrogel vascularisation. This indicates the potential to exquisitely modulate bone formation and architecture and the importance of incorporation of mechanotransduction cues. The hydrogel implants showed extensive host tissue invasion and vascularisation, demonstrating the potential for effective integration of tissue engineered constructs and *in vivo* defect repair. The nature of the hydrogel used was as such that these ALG/ECM microparticle constructs could be injectable, thereby providing clinicians with a minimally invasive reparative method for direct *in vivo* bone regeneration which can also be modulated with remote MNP mechano-regulation. The current results provide valuable data on the initial steps towards a platform shift from single-to multifaceted bone tissue engineering.

## Data availability

The datasets generated during and/or analysed during the current study are included in the article or available from the corresponding author on reasonable request.

## Ethical approval

Human bone marrow was collected following informed consent (approval from the Southampton and South West Hampshire Local Research Ethics Committee (LREC194/99)) from patients undergoing total hip-replacement surgery. All animal procedures were carried out in accordance with the guidelines and regulations laid down in the Animals (Scientific Procedures) Act 1986. MF1 nu/nu mice were sacrificed after 28 days using schedule 1 CO_2_ inhalation and cervical dislocation according to Home Office Approval UK (Project license – PPL 30/2762). All surgery was performed under anaesthesia/analgesia, and all efforts were made to minimise suffering.

## CRediT authorship contribution statement

**David Gothard:** Conceptualization, Formal analysis, Investigation, Methodology, Resources, Writing – original draft, Writing – review & editing. **Michael Rotherham:** Formal analysis, Investigation, Methodology, Resources, Writing – original draft, Writing – review & editing. **Emma L. Smith:** Conceptualization, Formal analysis, Investigation, Methodology, Resources, Writing – original draft, Writing – review & editing. **Janos M. Kanczler:** Conceptualization, Formal analysis, Investigation, Methodology, Resources, Writing – original draft, Writing – review & editing. **James Henstock:** Formal analysis, Investigation, Methodology, Resources, Writing – original draft, Writing – review & editing. **Julia A. Wells:** Formal analysis, Investigation, Methodology, Resources. **Carol A. Roberts:** Formal analysis, Investigation, Methodology, Resources. **Omar Qutachi:** Formal analysis, Investigation, Methodology, Resources, Writing – original draft, Writing – review & editing. **Heather Peto:** Formal analysis, Investigation, Methodology, Resources, Writing – original draft, Writing – review & editing. **Hassan Rashidi:** Formal analysis, Investigation, Methodology, Resources, Writing – original draft, Writing – review & editing. **Luis Rojo:** Formal analysis, Investigation, Methodology, Resources, Writing – original draft, Writing – review & editing. **Lisa J. White:** Formal analysis, Investigation, Methodology, Resources, Writing – original draft, Writing – review & editing. **Molly M. Stevens:** Conceptualization, Formal analysis, Funding acquisition, Resources, Supervision, Writing – original draft, Writing – review & editing. **Alicia J. El Haj:** Conceptualization, Formal analysis, Funding acquisition, Resources, Supervision, Writing – original draft, Writing – review & editing. **Felicity R.A.J. Rose:** Formal analysis, Resources, Supervision, Writing – original draft, Writing – review & editing. **Richard O.C. Oreffo:** Conceptualization, Formal analysis, Funding acquisition, Resources, Supervision, Writing – original draft, Writing – review & editing.

## Declaration of competing interest

The authors declare that they have no known competing financial interests or personal relationships that could have appeared to influence the work reported in this paper.
