# Supplementary material for: In vivo analysis of hybrid hydrogels containing dual growth factor combinations, and skeletal stem cells under mechanical stimulation for bone repair
Source: Mechanobiol Med. 2024 Aug 27;2(4):100096. doi: 10.1016/j.mbm.2024.100096 (PMC12082137; doi:10.1016/j.mbm.2024.100096)
Supplement: Multimedia component 1 [file mmc1.pdf]

Supplementary information

In vivo analysis of hybrid hydrogels containing dual growth factor combinations, and skeletal stem cells under mechanical stimulation for bone repair

David Gothard[a,1,2], Michael Rotherham[b,c,2,*], Emma L. Smith[a,1], Janos M. Kanczler[a], James Henstock[b,d], Julia A. Wells[a], Carol A. Roberts[a], Omar Qutachi[e,1,f], Heather Peto[e,1], Hassan Rashidi[e,1,g], Luis Rojo[h,i,1,j,k], Lisa J. White[l], Molly M. Stevens[h,i,m,1,n], Alicia J. El Haj[b,c], Felicity R. A. J. Rose[l] and Richard O. C. Oreffo[a**]

[a] Bone and Joint Research Group, Centre for Human Development, Stem Cells and Regeneration, Institute of Developmental Sciences, University of Southampton, Southampton, SO16 6YD, UK

[b] Institute for Science and Technology in Medicine, Keele University, Guy Hilton Research Centre, Stoke-on-Trent, ST4 7BQ, UK

[c] Healthcare Technologies Institute, School of Chemical Engineering, Institute of Translational Medicine, University of Birmingham, , Birmingham, B15 2TH, UK

[d] Department of Applied Sciences, Pandon Building, Northumbria University, Newcastle-upon-Tyne, NE2 1XE, UK

[e] Wolfson Centre for Stem Cells, Tissue Engineering and Modelling, University of Nottingham, Centre for Biomolecular Sciences, University Park, Nottingham, NG7 2RD, UK

[f] School of Pharmacy, Faculty of Health and Life Sciences, De Montfort University, The Gateway, Leicester, LE1 9BH, UK

[g] Stem Cells and Regenerative Medicine Section, UCL Great Ormond Street Institute of Child Health, University College London, London, WC1N 1EH, UK

[h] Department of Materials, Imperial College London, Royal School of Mines, London, SW7 2AZ, UK

[i] Department of Bioengineering, Imperial College London, South Kensington Campus, London, SW7 2AZ, UK

[j] Instituto de Ciencia y Tecnología de Polímeros (ICTP), CSIC, Calle Juan de la Cierva, 3, 28006 Madrid, Spain

[k] Consorcio Centro de Investigación Biomédica en Red, CIBER-BBN, Instituto de Salud Carlos III, Calle Monforte de Lemos 3–5, 1128029 Pabellón, Madrid, Spain

[l] Nottingham Biodiscovery Institute, School of Pharmacy, University of Nottingham, Nottingham, NG7 2RD, UK

[m] Institute for Biomedical Engineering Imperial College London, South Kensington Campus, London, SW7 2AZ, UK

[n] Department of Physiology, Anatomy, & Genetics, Department of Engineering Science, and Kavli Institute for Nanoscience Discovery, University of Oxford, Oxford, OX1 3QU, UK

[1] Affiliation at the time the studies were performed

[2] indicates joint authorship of the manuscript

* Correspondence to: Dr Michael Rotherham (m.rotherham@bham.ac.uk)

**Correspondence to: Professor Richard O. C. Oreffo (roco@soton.ac.uk)

Supplementary information

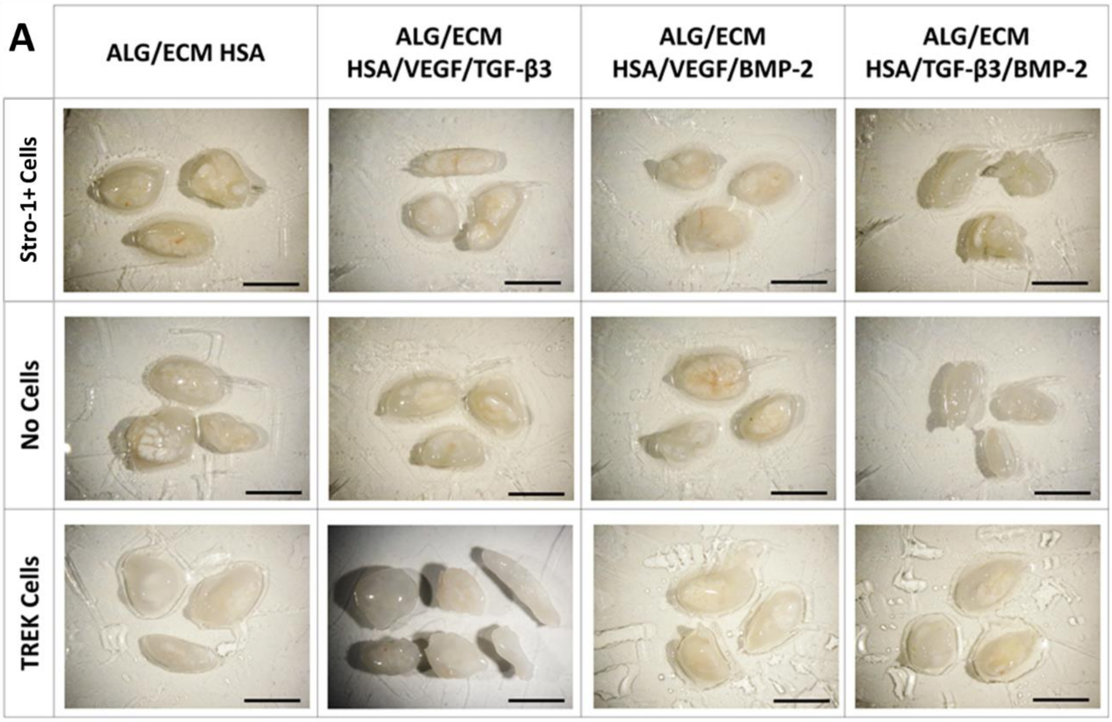

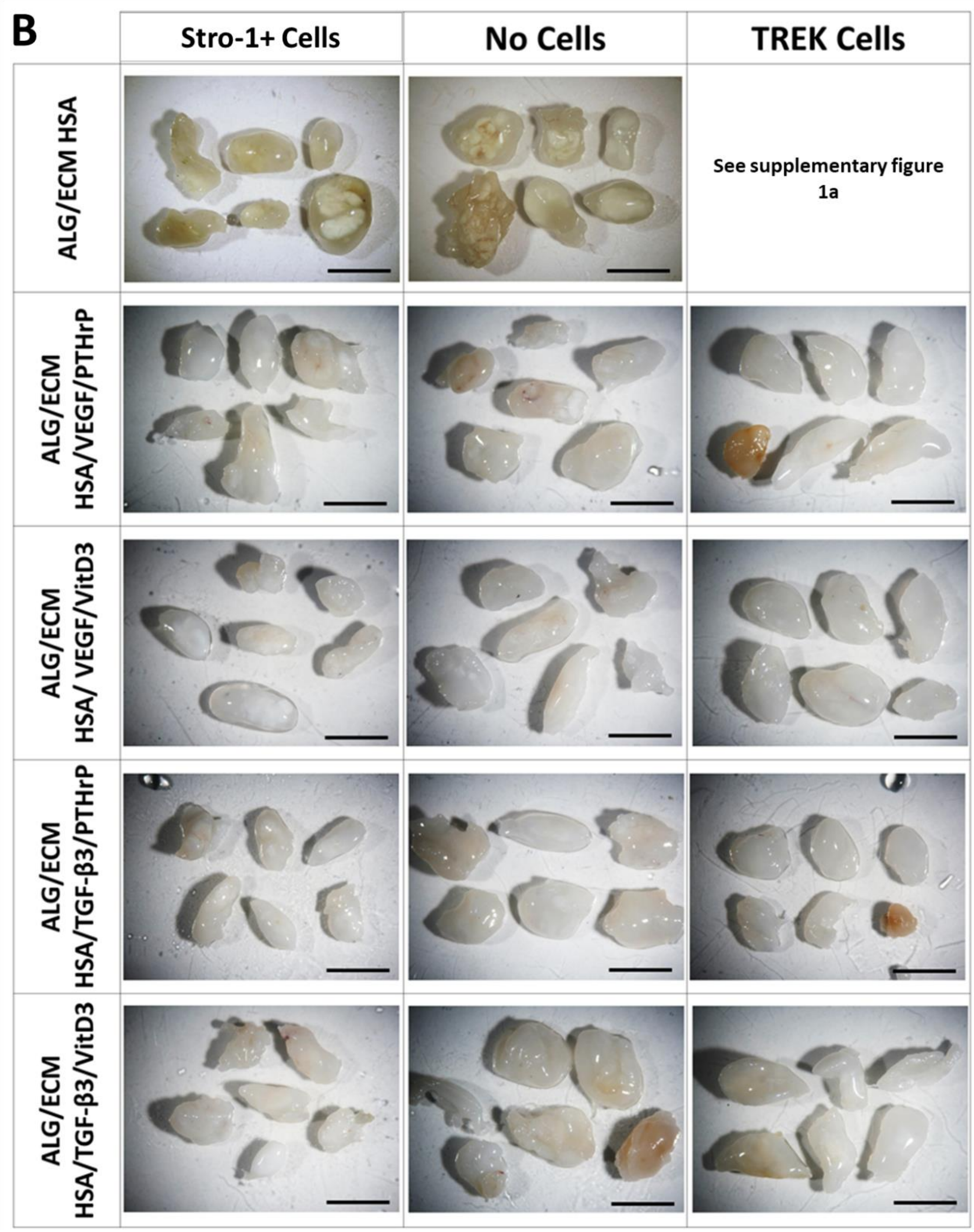

Supplementary figure 1: Hydrogels following harvest from immunodeficient mice after 28 days implantation. Scale bar is 5 mm. ALG/ECM, alginate, and bone extracellular matrix; HSA, human serum albumin; VEGF, vascular endothelial growth factor; TGF-β_3, transforming growth factor beta 3; BMP-2, bone morphogenetic protein 2; PTHrP, parathyroid hormone-related protein; VitD3, vitamin D3.

One way ANOVA (Tukeys) $p \le 0.05$ - **Bone Volume**

	Irradiated ALG / ECM	ALG / Col	ALG / ECM	ALG / ECM HSA	ALG / ECM HSA / VEGF / TGF-β3	ALG / ECM HSA / VEGF / BMP-2	ALG / ECM HSA / TGF-β3 / BMP-2
**Irradiated ALG / ECM**				NS	NS	NS	NS
**ALG / Col**				NS	NS	NS	NS
**ALG / ECM**				NS	NS	NS	*
**ALG / ECM HSA**	NS	NS	NS		NS	NS	NS
**ALG / ECM HSA / VEGF / TGF-β3**	NS	NS	NS	NS		NS	NS
**ALG / ECM HSA / VEGF / BMP-2**	NS	NS	NS	NS	NS		NS
**ALG / ECM HSA / TGF - β3 / BMP-2**	NS	NS	NS	NS	NS	NS	

Tissue Volume - *One way ANOVA (Tukeys) $p > 0.05$*

One way ANOVA (Tukeys) $p \le 0.01$ - **Percentage Bone Volume**

	Irradiated ALG / ECM	ALG / Col	ALG / ECM	ALG / ECM HSA	ALG / ECM HSA / VEGF / TGF-β3	ALG / ECM HSA / VEGF / BMP-2	ALG / ECM HSA / TGF-β3 / BMP-2
**Irradiated ALG / ECM**				NS	NS	NS	NS
**ALG / Col**				NS	NS	NS	NS
**ALG / ECM**				NS	NS	NS	**
**ALG / ECM HSA**	NS	NS	NS		NS	NS	NS
**ALG / ECM HSA / VEGF / TGF-β3**	NS	NS	NS	NS		NS	NS
**ALG / ECM HSA / VEGF / BMP-2**	NS	NS	NS	NS	NS		NS
**ALG / ECM HSA / TGF - β3 / BMP-2**	NS	NS	NS	NS	NS	NS	

Bone Surface/Volume Ratio - *One way ANOVA (Tukeys) $p > 0.05$*

One way ANOVA (Tukeys) $p > 0.05$ - **Trabecular Number**

	Irradiated ALG / ECM	ALG / Col	ALG / ECM	ALG / ECM HSA	ALG / ECM HSA / VEGF / TGF-β3	ALG / ECM HSA / VEGF / BMP-2	ALG / ECM HSA / TGF-β3 / BMP-2
**Irradiated ALG / ECM**				NS	NS	NS	NS
**ALG / Col**				NS	NS	NS	NS
**ALG / ECM**				NS	NS	NS	NS
**ALG / ECM HSA**	NS	NS	NS		NS	NS	NS
**ALG / ECM HSA / VEGF / TGF-β3**	NS	NS	NS	NS		NS	NS
**ALG / ECM HSA / VEGF / BMP-2**	NS	NS	NS	NS	NS		NS
**ALG / ECM HSA / TGF - β3 / BMP-2**	NS	NS	*	NS	NS	NS	

Trabecular Thickness - *One way ANOVA (Tukeys) $p \le 0.05$*

One way ANOVA (Tukeys) $p > 0.05$ - **Trabecular Seperation**

	Irradiated ALG / ECM	ALG / Col	ALG / ECM	ALG / ECM HSA	ALG / ECM HSA / VEGF / TGF-β3	ALG / ECM HSA / VEGF / BMP-2	ALG / ECM HSA / TGF-β3 / BMP-2
**Irradiated ALG / ECM**				NS	NS	NS	NS
**ALG / Col**				NS	NS	NS	NS
**ALG / ECM**				NS	NS	NS	NS
**ALG / ECM HSA**					NS	NS	NS
**ALG / ECM HSA / VEGF / TGF-β3**						NS	NS
**ALG / ECM HSA / VEGF / BMP-2**							NS
**ALG / ECM HSA / TGF - β3 / BMP-2**							

One way ANOVA (Tukeys) p ≤ 0.001 - **Bone Volume**

	Irradiated ALG / ECM	ALG / Col	ALG / ECM	ALG / ECM HSA	ALG / ECM HSA / VEGF / PTHrP	ALG / ECM HSA / VEGF / VitD3	ALG / ECM HSA / TGF - β3 / PTHrP	ALG / ECM HSA / TGF - β3 / VitD3
Irradiated ALG / ECM				NS	NS	NS	NS	NS
ALG / Col				NS	NS	NS	NS	NS
ALG / ECM				NS	**	**	**	**
ALG / ECM HSA	NS	NS	NS		NS	NS	NS	NS
ALG / ECM HSA / VEGF / PTHrP	NS	NS	NS	NS		NS	NS	NS
ALG / ECM HSA / VEGF / VitD3	NS	NS	NS	NS	NS		NS	NS
ALG / ECM HSA / TGF - β3 / PTHrP	NS	NS	NS	NS	NS	NS		NS
ALG / ECM HSA / TGF - β3 / VitD3	NS	NS	NS	NS	NS	NS	NS	

Tissue Volume - *One way ANOVA (Tukeys) p ≤ 0.05*

One way ANOVA (Tukeys) p ≤ 0.001 - **Percentage Bone Volume**

	Irradiated ALG / ECM	ALG / Col	ALG / ECM	ALG / ECM HSA	ALG / ECM HSA / VEGF / PTHrP	ALG / ECM HSA / VEGF / VitD3	ALG / ECM HSA / TGF - β3 / PTHrP	ALG / ECM HSA / TGF - β3 / VitD3
Irradiated ALG / ECM				*	NS	NS	NS	NS
ALG / Col				NS	NS	NS	NS	NS
ALG / ECM				NS	***	***	***	***
ALG / ECM HSA	NS	NS	NS		**	**	**	**
ALG / ECM HSA / VEGF / PTHrP	NS	NS	NS	NS		NS	NS	NS
ALG / ECM HSA / VEGF / VitD3	NS	NS	NS	NS	NS		NS	NS
ALG / ECM HSA / TGF - β3 / PTHrP	**	**	***	***	**	*		NS
ALG / ECM HSA / TGF - β3 / VitD3	*	*	**	**	NS	NS	NS	

Bone Surface/Volume Ratio - *One way ANOVA (Tukeys) p ≤ 0.001*

One way ANOVA (Tukeys) p ≤ 0.001 - **Trabecular Number**

	Irradiated ALG / ECM	ALG / Col	ALG / ECM	ALG / ECM HSA	ALG / ECM HSA / VEGF / PTHrP	ALG / ECM HSA / VEGF / VitD3	ALG / ECM HSA / TGF - β3 / PTHrP	ALG / ECM HSA / TGF - β3 / VitD3
Irradiated ALG / ECM				NS	NS	NS	**	**
ALG / Col				NS	NS	NS	**	**
ALG / ECM				NS	NS	NS	***	***
ALG / ECM HSA	NS	NS	NS		NS	NS	***	***
ALG / ECM HSA / VEGF / PTHrP	NS	*	***	*		NS	*	*
ALG / ECM HSA / VEGF / VitD3	NS	*	***	*	NS		*	*
ALG / ECM HSA / TGF - β3 / PTHrP	**	***	***	***	NS	NS		NS
ALG / ECM HSA / TGF - β3 / VitD3	**	***	***	***	NS	NS	NS	

Trabecular Thickness - *One way ANOVA (Tukeys) p ≤ 0.001*

One way ANOVA (Tukeys) p > 0.05 - **Trabecular Seperation**

	Irradiated ALG / ECM	ALG / Col	ALG / ECM	ALG / ECM HSA	ALG / ECM HSA / VEGF / PTHrP	ALG / ECM HSA / VEGF / VitD3	ALG / ECM HSA / TGF - β3 / PTHrP	ALG / ECM HSA / TGF - β3 / VitD3
Irradiated ALG / ECM				NS	NS	NS	NS	NS
ALG / Col				NS	NS	NS	NS	NS
ALG / ECM				NS	NS	NS	NS	NS
ALG / ECM HSA					NS	NS	NS	NS
ALG / ECM HSA / VEGF / PTHrP						NS	NS	NS
ALG / ECM HSA / VEGF / VitD3							NS	NS
ALG / ECM HSA / TGF - β3 / PTHrP								NS
ALG / ECM HSA / TGF - β3 / VitD3								

Supplementary figure 2: Statistical analysis of micro-CT data between growth/osteoinductive factor groups without Stro-1+ cell incorporation. All data was analysed using one way ANOVA with Tukeys post-hoc test. Tables separate into upper right and lower left corners detailing individual comparisons between all groups regarding the parameter stated adjacent. For comparisons between control groups, please refer to Gothard D et al. 2015 [34]. NS indicates 'no significance'. * P ≤ 0.05, ** P ≤ 0.01, *** P ≤ 0.001.

ALG/ECM, alginate, and bone extracellular matrix; HSA, human serum albumin; VEGF, vascular endothelial growth factor; TGF-β_3, transforming growth factor beta 3; BMP-2, bone morphogenetic protein 2 PTHrP, parathyroid hormone-related protein; VitD3, vitamin D_3.

One way ANOVA (Tukeys) $p > 0.05$ - **Bone Volume**

	Irradiated ALG / ECM	ALG / Col	ALG / ECM	ALG / ECM HSA	ALG / ECM HSA / VEGF / TGF-β3	ALG / ECM HSA / VEGF / BMP-2	ALG / ECM HSA / TGF-β3 / BMP-2
**Irradiated ALG / ECM**				**NS**	**NS**	**NS**	**NS**
**ALG / Col**				**NS**	**NS**	**NS**	**NS**
**ALG / ECM**				**NS**	**NS**	**NS**	**NS**
**ALG / ECM HSA**	**NS**	**NS**	**NS**		**NS**	**NS**	**NS**
**ALG / ECM HSA / VEGF / TGF-β3**	**NS**	**NS**	**NS**	**NS**		**NS**	**NS**
**ALG / ECM HSA / VEGF / BMP-2**	**NS**	**NS**	**NS**	**NS**	**NS**		**NS**
**ALG / ECM HSA / TGF - β3 / BMP-2**	**NS**	**NS**	**NS**	**NS**	**NS**	**NS**	

Tissue Volume - *One way ANOVA (Tukeys) $p > 0.05$*

One way ANOVA (Tukeys) $p \leq 0.05$ - **Percentage Bone Volume**

	Irradiated ALG / ECM	ALG / Col	ALG / ECM	ALG / ECM HSA	ALG / ECM HSA / VEGF / TGF-β3	ALG / ECM HSA / VEGF / BMP-2	ALG / ECM HSA / TGF-β3 / BMP-2
**Irradiated ALG / ECM**				**NS**	**NS**	**NS**	**NS**
**ALG / Col**				**NS**	**NS**	**NS**	**NS**
**ALG / ECM**				**NS**	**NS**	**NS**	**NS**
**ALG / ECM HSA**	**NS**	**NS**	**NS**		**NS**	**NS**	**NS**
**ALG / ECM HSA / VEGF / TGF-β3**	**NS**	**NS**	**NS**	**NS**		**NS**	**NS**
**ALG / ECM HSA / VEGF / BMP-2**	**NS**	**NS**	**NS**	**NS**	**NS**		**NS**
**ALG / ECM HSA / TGF - β3 / BMP-2**	**NS**	**NS**	**NS**	**NS**	**NS**	**NS**	

Bone Surface/Volume Ratio - *One way ANOVA (Tukeys) $p > 0.05$*

One way ANOVA (Tukeys) $p > 0.05$ - **Trabecular Number**

	Irradiated ALG / ECM	ALG / Col	ALG / ECM	ALG / ECM HSA	ALG / ECM HSA / VEGF / TGF-β3	ALG / ECM HSA / VEGF / BMP-2	ALG / ECM HSA / TGF-β3 / BMP-2
**Irradiated ALG / ECM**				**NS**	**NS**	**NS**	**NS**
**ALG / Col**				**NS**	**NS**	**NS**	**NS**
**ALG / ECM**				**NS**	**NS**	**NS**	**NS**
**ALG / ECM HSA**	**NS**	**NS**	**NS**		**NS**	**NS**	**NS**
**ALG / ECM HSA / VEGF / TGF-β3**	**NS**	**NS**	**NS**	**NS**		**NS**	**NS**
**ALG / ECM HSA / VEGF / BMP-2**	**NS**	**NS**	**NS**	**NS**	**NS**		**NS**
**ALG / ECM HSA / TGF - β3 / BMP-2**	**NS**	**NS**	**NS**	**NS**	**NS**	**NS**	

Trabecular Thickness - *One way ANOVA (Tukeys) $p \leq 0.05$*

One way ANOVA (Tukeys) $p > 0.05$ - **Trabecular Seperation**

	Irradiated ALG / ECM	ALG / Col	ALG / ECM	ALG / ECM HSA	ALG / ECM HSA / VEGF / TGF-β3	ALG / ECM HSA / VEGF / BMP-2	ALG / ECM HSA / TGF-β3 / BMP-2
**Irradiated ALG / ECM**				**NS**	**NS**	**NS**	**NS**
**ALG / Col**				**NS**	**NS**	**NS**	**NS**
**ALG / ECM**				**NS**	**NS**	**NS**	**NS**
**ALG / ECM HSA**					**NS**	**NS**	**NS**
**ALG / ECM HSA / VEGF / TGF-β3**						**NS**	**NS**
**ALG / ECM HSA / VEGF / BMP-2**							**NS**
**ALG / ECM HSA / TGF - β3 / BMP-2**							

One way ANOVA (Tukeys) p ≤ 0.001 - **Bone Volume**

	Irradiated ALG / ECM	ALG / Col	ALG / ECM	ALG / ECM HSA	ALG / ECM HSA / VEGF / PTHrP	ALG / ECM HSA / VEGF / VitD3	ALG / ECM HSA / TGF - β3 / PTHrP	ALG / ECM HSA / TGF - β3 / VitD3
Irradiated ALG / ECM				NS	NS	NS	NS	NS
ALG / Col				NS	NS	NS	NS	NS
ALG / ECM				*	**	**	**	**
ALG / ECM HSA	NS	NS	NS		NS	NS	NS	NS
ALG / ECM HSA / VEGF / PTHrP	NS	NS	NS	NS		NS	NS	NS
ALG / ECM HSA / VEGF / VitD3	NS	NS	NS	NS	NS		NS	NS
ALG / ECM HSA / TGF - β3 / PTHrP	NS	NS	NS	NS	NS	NS		NS
ALG / ECM HSA / TGF - β3 / VitD3	NS	NS	NS	NS	NS	NS	NS	

Tissue Volume - *One way ANOVA (Tukeys) p ≤ 0.05*

One way ANOVA (Tukeys) p ≤ 0.001 - **Percentage Bone Volume**

	Irradiated ALG / ECM	ALG / Col	ALG / ECM	ALG / ECM HSA	ALG / ECM HSA / VEGF / PTHrP	ALG / ECM HSA / VEGF / VitD3	ALG / ECM HSA / TGF - β3 / PTHrP	ALG / ECM HSA / TGF - β3 / VitD3
Irradiated ALG / ECM				NS	NS	NS	NS	NS
ALG / Col				NS	NS	NS	NS	NS
ALG / ECM				*	***	*	**	**
ALG / ECM HSA	NS	NS	NS		NS	NS	NS	NS
ALG / ECM HSA / VEGF / PTHrP	*	***	***	***		NS	NS	NS
ALG / ECM HSA / VEGF / VitD3	NS	NS	NS	NS	NS		NS	NS
ALG / ECM HSA / TGF - β3 / PTHrP	NS	NS	NS	NS	**	NS		NS
ALG / ECM HSA / TGF - β3 / VitD3	NS	**	***	***	NS	NS	NS	

Bone Surface/Volume Ratio - *One way ANOVA (Tukeys) p ≤ 0.001*

One way ANOVA (Tukeys) p ≤ 0.001 - **Trabecular Number**

	Irradiated ALG / ECM	ALG / Col	ALG / ECM	ALG / ECM HSA	ALG / ECM HSA / VEGF / PTHrP	ALG / ECM HSA / VEGF / VitD3	ALG / ECM HSA / TGF - β3 / PTHrP	ALG / ECM HSA / TGF - β3 / VitD3
Irradiated ALG / ECM				NS	**	NS	NS	NS
ALG / Col				NS	***	NS	NS	**
ALG / ECM				NS	***	NS	NS	**
ALG / ECM HSA	NS	NS	NS		***	NS	NS	**
ALG / ECM HSA / VEGF / PTHrP	**	***	***	***		*	**	NS
ALG / ECM HSA / VEGF / VitD3	NS	**	***	**	NS		NS	NS
ALG / ECM HSA / TGF - β3 / PTHrP	NS	**	**	*	NS	NS		NS
ALG / ECM HSA / TGF - β3 / VitD3	*	***	***	***	NS	NS	NS	

Trabecular Thickness - *One way ANOVA (Tukeys) p ≤ 0.001*

One way ANOVA (Tukeys) p > 0.05 - **Trabecular Seperation**

	Irradiated ALG / ECM	ALG / Col	ALG / ECM	ALG / ECM HSA	ALG / ECM HSA / VEGF / PTHrP	ALG / ECM HSA / VEGF / VitD3	ALG / ECM HSA / TGF - β3 / PTHrP	ALG / ECM HSA / TGF - β3 / VitD3
Irradiated ALG / ECM				NS	NS	NS	NS	NS
ALG / Col				NS	NS	NS	NS	NS
ALG / ECM				NS	NS	NS	NS	NS
ALG / ECM HSA					NS	NS	NS	NS
ALG / ECM HSA / VEGF / PTHrP						NS	NS	NS
ALG / ECM HSA / VEGF / VitD3							NS	NS
ALG / ECM HSA / TGF - β3 / PTHrP								NS
ALG / ECM HSA / TGF - β3 / VitD3								

Supplementary figure 3: Statistical analysis of micro-CT data between growth/osteoinductive factor groups with Stro-1+ cell incorporation. All data was analysed using one-way ANOVA with Tukeys post-hoc test. Tables separate into upper right and lower left corners detailing individual comparisons between all groups regarding the parameter stated adjacent. For comparisons between control groups, please refer to Gothard D et al. 2015 [34]. NS indicates ‘no significance’. * P ≤ 0.05, ** P ≤ 0.01, *** P ≤ 0.001.

ALG/ECM, alginate, and bone extracellular matrix; HSA, human serum albumin; VEGF, vascular endothelial growth factor; TGF-β_3, transforming growth factor beta 3; BMP-2, bone morphogenetic protein 2; PTHrP, parathyroid hormone-related protein; VitD3, vitamin D3.

One way ANOVA (Tukeys) $p \leq 0.01$ - **Bone Volume**

	ALG / ECM HSA	ALG / ECM HSA / VEGF / TGF-β3	ALG / ECM HSA / VEGF / BMP-2	ALG / ECM HSA / TGF-β3 / BMP-2
**ALG / ECM HSA**		NS	NS	NS
**ALG / ECM HSA / VEGF / TGF-β3**	NS		*	*
**ALG / ECM HSA / VEGF / BMP-2**	NS	NS		NS
**ALG / ECM HSA / TGF-β3 / BMP-2**	NS	NS	NS	

Tissue Volume - *One way ANOVA (Tukeys) $p > 0.05$*

One way ANOVA (Tukeys) $p \leq 0.05$ - **Percentage Bone Volume**

	ALG / ECM HSA	ALG / ECM HSA / VEGF / TGF-β3	ALG / ECM HSA / VEGF / BMP-2	ALG / ECM HSA / TGF-β3 / BMP-2
**ALG / ECM HSA**		NS	NS	NS
**ALG / ECM HSA / VEGF / TGF-β3**	**		NS	*
**ALG / ECM HSA / VEGF / BMP-2**	NS	***		NS
**ALG / ECM HSA / TGF-β3 / BMP-2**	NS	***	NS	

Bone Surface/Volume Ratio - *One way ANOVA (Tukeys) $p \leq 0.001$*

One way ANOVA (Tukeys) $p \leq 0.01$ - **Trabecular Number**

	ALG / ECM HSA	ALG / ECM HSA / VEGF / TGF-β3	ALG / ECM HSA / VEGF / BMP-2	ALG / ECM HSA / TGF-β3 / BMP-2
**ALG / ECM HSA**		NS	NS	NS
**ALG / ECM HSA / VEGF / TGF-β3**	*		**	**
**ALG / ECM HSA / VEGF / BMP-2**	NS	***		NS
**ALG / ECM HSA / TGF-β3 / BMP-2**	NS	***	NS	

Trabecular Thickness - *One way ANOVA (Tukeys) $p \leq 0.001$*

One way ANOVA (Tukeys) $p > 0.05$ - **Trabecular Seperation**

	ALG / ECM HSA	ALG / ECM HSA / VEGF / TGF-β3	ALG / ECM HSA / VEGF / BMP-2	ALG / ECM HSA / TGF-β3 / BMP-2
**ALG / ECM HSA**		NS	NS	NS
**ALG / ECM HSA / VEGF / TGF-β3**			NS	NS
**ALG / ECM HSA / VEGF / BMP-2**				NS
**ALG / ECM HSA / TGF-β3 / BMP-2**				

One way ANOVA (Tukeys) p ≤ 0.001 - **Bone Volume**

	ALG / ECM HSA	ALG / ECM HSA / VEGF / PTHrP	ALG / ECM HSA / VEGF / VitD3	ALG / ECM HSA / TGF - β3 / PTHrP	ALG / ECM HSA / TGF - β3 / VitD3
ALG / ECM HSA		***	***	***	*
ALG / ECM HSA / VEGF / PTHrP	NS		NS	NS	NS
ALG / ECM HSA / VEGF / VitD3	NS	NS		NS	NS
ALG / ECM HSA / TGF - β3 / PTHrP	NS	NS	NS		NS
ALG / ECM HSA / TGF - β3 / VitD3	NS	NS	NS	NS	

Tissue Volume - *One way ANOVA (Tukeys) p > 0.05*

One way ANOVA (Tukeys) p ≤ 0.001 - **Percentage Bone Volume**

	ALG / ECM HSA	ALG / ECM HSA / VEGF / PTHrP	ALG / ECM HSA / VEGF / VitD3	ALG / ECM HSA / TGF - β3 / PTHrP	ALG / ECM HSA / TGF - β3 / VitD3
ALG / ECM HSA		***	***	***	**
ALG / ECM HSA / VEGF / PTHrP	NS		NS	NS	NS
ALG / ECM HSA / VEGF / VitD3	NS	NS		NS	NS
ALG / ECM HSA / TGF - β3 / PTHrP	NS	NS	NS		NS
ALG / ECM HSA / TGF - β3 / VitD3	NS	NS	NS	NS	

Bone Surface/Volume Ratio - *One way ANOVA (Tukeys) p > 0.05*

One way ANOVA (Tukeys) p > 0.05 - **Trabecular Number**

	ALG / ECM HSA	ALG / ECM HSA / VEGF / PTHrP	ALG / ECM HSA / VEGF / VitD3	ALG / ECM HSA / TGF - β3 / PTHrP	ALG / ECM HSA / TGF - β3 / VitD3
ALG / ECM HSA		NS	NS	NS	NS
ALG / ECM HSA / VEGF / PTHrP	NS		NS	NS	NS
ALG / ECM HSA / VEGF / VitD3	*	NS		NS	NS
ALG / ECM HSA / TGF - β3 / PTHrP	***	**	**		NS
ALG / ECM HSA / TGF - β3 / VitD3	NS	NS	NS	***	

Trabecular Thickness - *One way ANOVA (Tukeys) p ≤ 0.001*

One way ANOVA (Tukeys) p ≤ 0.001 - **Trabecular Seperation**

	ALG / ECM HSA	ALG / ECM HSA / VEGF / PTHrP	ALG / ECM HSA / VEGF / VitD3	ALG / ECM HSA / TGF - β3 / PTHrP	ALG / ECM HSA / TGF - β3 / VitD3
ALG / ECM HSA		NS	NS	***	NS
ALG / ECM HSA / VEGF / PTHrP			NS	NS	NS
ALG / ECM HSA / VEGF / VitD3				NS	NS
ALG / ECM HSA / TGF - β3 / PTHrP					*
ALG / ECM HSA / TGF - β3 / VitD3					

Supplementary figure 4: Statistical analysis of micro-CT data between growth factor groups with TREK cell incorporation. All data was analysed using one-way ANOVA with Tukeys post-hoc test. Tables separate into upper right and lower left corners detailing individual comparisons between all groups regarding the parameter stated adjacent. NS indicates 'no significance'. * P ≤ 0.05, ** P ≤ 0.01, *** P ≤ 0.001.

ALG/ECM, alginate, and bone extracellular matrix; HSA, human serum albumin; VEGF, vascular endothelial growth factor; TGF-β_3, transforming growth factor beta 3; BMP-2, bone morphogenetic protein 2; PTHrP, parathyroid hormone-related protein; VitD3, vitamin D_3.

One way ANOVA (Tukeys) - **Intra-group significance**

		Bone Volume		Tissue Volume		Percentage Bone Volume		Bone Surface / Volume Ratio	
		One way ANOVA	Tukeys	One way ANOVA	Tukeys	One way ANOVA	Tukeys	One way ANOVA	Tukeys
ALG / ECM HSA	Stro-1+ cells / No cells		NS		NS		NS		NS
	Stro-1+ cells / TREK	P > 0.05	NS	P > 0.05	NS	P > 0.05	NS	P > 0.05	NS
	No cells / TREK		NS		NS		NS		NS
ALG / ECM HSA / VEGF / TGF-β3	Stro-1+ cells / No cells		NS		NS		NS		NS
	Stro-1+ cells / TREK	P > 0.05	NS	P > 0.05	NS	P > 0.05	NS	P ≤ 0.01	*
	No cells / TREK		NS		NS		NS		*
ALG / ECM HSA / VEGF / BMP-2	Stro-1+ cells / No cells		NS		NS		NS		NS
	Stro-1+ cells / TREK	P > 0.05	NS	P > 0.05	NS	P > 0.05	NS	P > 0.05	NS
	No cells / TREK		NS		NS		NS		NS
ALG / ECM HSA / TGF-β3 / BMP-2	Stro-1+ cells / No cells		NS		NS		NS		NS
	Stro-1+ cells / TREK	P ≤ 0.01	*	P > 0.05	NS	P ≤ 0.01	**	P ≤ 0.05	NS
	No cells / TREK		**		NS		**		NS

One way ANOVA (Tukeys) - **Intra-group significance continued**

		Trabecular Number		Trabecular Thickness		Trabecular Seperation	
		One way ANOVA	Tukeys	One way ANOVA	Tukeys	One way ANOVA	Tukeys
ALG / ECM HSA	Stro-1+ cells / No cells		NS		NS		NS
	Stro-1+ cells / TREK	P > 0.05	NS	P > 0.05	NS	P > 0.05	NS
	No cells / TREK		NS		NS		NS
ALG / ECM HSA / VEGF / TGF-β3	Stro-1+ cells / No cells		NS		NS		NS
	Stro-1+ cells / TREK	P ≤ 0.05	NS	P ≤ 0.01	*	P > 0.05	NS
	No cells / TREK		*		*		NS
ALG / ECM HSA / VEGF / BMP-2	Stro-1+ cells / No cells		NS		NS		NS
	Stro-1+ cells / TREK	P > 0.05	NS	P > 0.05	NS	P > 0.05	NS
	No cells / TREK		NS		NS		NS
ALG / ECM HSA / TGF-β3 / BMP-2	Stro-1+ cells / No cells		NS		NS		NS
	Stro-1+ cells / TREK	P > 0.05	NS	P ≤ 0.05	NS	P > 0.05	NS
	No cells / TREK		NS		*		NS

One way ANOVA (Tukeys) - **Intra-group significance**

		Bone Volume		Tissue Volume		Percentage Bone Volume		Bone Surface / Volume Ratio	
		One way ANOVA	Tukeys	One way ANOVA	Tukeys	One way ANOVA	Tukeys	One way ANOVA	Tukeys
ALG / ECM HSA	Stro-1+ cells / No cells		NS		NS		NS		NS
	Stro-1+ cells / TREK	P > 0.05	NS	P > 0.05	NS	P > 0.05	NS	P > 0.05	NS
	No cells / TREK		NS		NS		NS		NS
ALG / ECM HSA / VEGF / PTHrP	Stro-1+ cells / No cells		NS		NS		NS		NS
	Stro-1+ cells / TREK	P > 0.05	NS	P > 0.05	NS	P > 0.05	NS	P > 0.05	NS
	No cells / TREK		NS		NS		NS		NS
ALG / ECM HSA / VEGF / VitD3	Stro-1+ cells / No cells		NS		**		NS		NS
	Stro-1+ cells / TREK	P > 0.05	NS	P ≤ 0.01	**	P > 0.05	NS	P ≤ 0.001	***
	No cells / TREK		NS		NS		NS		**
ALG / ECM HSA / TGF-β3 / PTHrP	Stro-1+ cells / No cells		*		NS		**		NS
	Stro-1+ cells / TREK	P ≤ 0.01	**	P ≤ 0.05	*	P ≤ 0.001	**	P ≤ 0.05	NS
	No cells / TREK		NS		NS		NS		*
ALG / ECM HSA / TGF-β3 / VitD3	Stro-1+ cells / No cells		NS		**		*		**
	Stro-1+ cells / TREK	P > 0.05	NS	P ≤ 0.01	NS	P ≤ 0.05	NS	P ≤ 0.01	NS
	No cells / TREK		NS		*		NS		**

One way ANOVA (Tukeys) - **Intra-group significance continued**

		Trabecular Number		Trabecular Thickness		Trabecular Seperation	
		One way ANOVA	Tukeys	One way ANOVA	Tukeys	One way ANOVA	Tukeys
ALG / ECM HSA	Stro-1+ cells / No cells		NS		NS		NS
	Stro-1+ cells / TREK	P ≤ 0.05	*	P > 0.05	NS	P > 0.05	NS
	No cells / TREK		NS		NS		NS
ALG / ECM HSA / VEGF / PTHrP	Stro-1+ cells / No cells		NS		NS		NS
	Stro-1+ cells / TREK	P > 0.05	NS	P > 0.05	NS	P > 0.05	NS
	No cells / TREK		NS		NS		NS
ALG / ECM HSA / VEGF / VitD3	Stro-1+ cells / No cells		NS		NS		NS
	Stro-1+ cells / TREK	P ≤ 0.001	***	P ≤ 0.001	***	P > 0.05	NS
	No cells / TREK		**		**		NS
ALG / ECM HSA / TGF-β3 / PTHrP	Stro-1+ cells / No cells		NS		***		**
	Stro-1+ cells / TREK	P ≤ 0.05	NS	P ≤ 0.001	***	P ≤ 0.001	***
	No cells / TREK		*		**		NS
ALG / ECM HSA / TGF-β3 / VitD3	Stro-1+ cells / No cells		*		**		NS
	Stro-1+ cells / TREK	P ≤ 0.01	NS	P ≤ 0.001	NS	P > 0.05	NS
	No cells / TREK		**		***		NS

Supplementary figure 5: Statistical analysis of micro-CT data between those groups with Stro-1+ cell, without cell, and with TREK cell incorporation. NS indicates 'no significance.' * P ≤ 0.05, ** P ≤ 0.01, *** P ≤ 0.001.

ALG/ECM, alginate, and bone extracellular matrix; HSA, human serum albumin; VEGF, vascular endothelial growth factor; TGF-β_3, transforming growth factor beta 3; BMP-2, bone morphogenetic protein 2; PTHrP, parathyroid hormone-related protein; VitD3, vitamin D3.

One way ANOVA (Tukeys) p ≤ 0.01 - **Stro-1+ cells**

Residual Hydrogel and Proteoglycan	Irradiated ALG / ECM	ALG / Col	ALG / ECM	ALG / ECM HSA	ALG / ECM HSA / VEGF / TGF-β3	ALG / ECM HSA / VEGF / BMP-2	ALG / ECM HSA / TGF-β3 / BMP-2
Irradiated ALG / ECM				**	NS	*	*
ALG / Col				NS	NS	NS	NS
ALG / ECM				NS	NS	NS	NS
ALG / ECM HSA	NS	NS	NS		NS	NS	NS
ALG / ECM HSA / VEGF / TGF-β3	NS	NS	***	NS		NS	NS
ALG / ECM HSA / VEGF / BMP-2	NS	NS	NS	NS	NS		NS
ALG / ECM HSA / TGF - β3 / BMP-2	NS	NS	NS	NS	NS	NS	

No cells - *One way ANOVA (Tukeys) p ≤ 0.001*

One way ANOVA (Tukeys) p ≤ 0.001 - **Stro-1+ cells**

Collagen Deposition	Irradiated ALG / ECM	ALG / Col	ALG / ECM	ALG / ECM HSA	ALG / ECM HSA / VEGF / TGF-β3	ALG / ECM HSA / VEGF / BMP-2	ALG / ECM HSA / TGF-β3 / BMP-2
Irradiated ALG / ECM				NS	NS	***	NS
ALG / Col				NS	NS	***	NS
ALG / ECM				NS	NS	***	NS
ALG / ECM HSA	**	*	**		NS	***	NS
ALG / ECM HSA / VEGF / TGF-β3	NS	NS	NS	NS		***	NS
ALG / ECM HSA / VEGF / BMP-2	NS	NS	NS	NS	NS		**
ALG / ECM HSA / TGF - β3 / BMP-2	NS	NS	NS	NS	NS	NS	

No cells - *One way ANOVA (Tukeys) p ≤ 0.01*

One way ANOVA (Tukeys) p ≤ 0.001 - **Stro-1+ cells**

Tissue Invasion	Irradiated ALG / ECM	ALG / Col	ALG / ECM	ALG / ECM HSA	ALG / ECM HSA / VEGF / TGF-β3	ALG / ECM HSA / VEGF / BMP-2	ALG / ECM HSA / TGF-β3 / BMP-2
Irradiated ALG / ECM				NS	NS	*	***
ALG / Col				NS	NS	NS	**
ALG / ECM				NS	NS	*	***
ALG / ECM HSA	NS	NS	NS		NS	NS	**
ALG / ECM HSA / VEGF / TGF-β3	**	NS	**	NS		NS	NS
ALG / ECM HSA / VEGF / BMP-2	**	NS	**	NS	NS		NS
ALG / ECM HSA / TGF - β3 / BMP-2	*	NS	*	NS	NS	NS	

No cells - *One way ANOVA (Tukeys) p ≤ 0.001*

One way ANOVA (Tukeys) p ≤ 0.01 - **Residual Hydrogel and Proteoglycan**

TREK Cells	Irradiated ALG / ECM	ALG / Col	ALG / ECM	ALG / ECM HSA	ALG / ECM HSA / VEGF / TGF-β3	ALG / ECM HSA / VEGF / BMP-2	ALG / ECM HSA / TGF-β3 / BMP-2
Irradiated ALG / ECM				NS	NS	*	NS
ALG / Col				NS	NS	NS	NS
ALG / ECM				NS	NS	NS	NS
ALG / ECM HSA	*	NS	NS		NS	NS	NS
ALG / ECM HSA / VEGF / TGF-β3	*	NS	**	NS		NS	NS
ALG / ECM HSA / VEGF / BMP-2	*	NS	NS	NS	NS		NS
ALG / ECM HSA / TGF - β3 / BMP-2	***	NS	NS	NS	NS	NS	

Collagen Deposition - *One way ANOVA (Tukeys) p ≤ 0.001*

One way ANOVA (Tukeys) p ≤ 0.001 - **Tissue Invasion**

TREK Cells	Irradiated ALG / ECM	ALG / Col	ALG / ECM	ALG / ECM HSA	ALG / ECM HSA / VEGF / TGF-β3	ALG / ECM HSA / VEGF / BMP-2	ALG / ECM HSA / TGF-β3 / BMP-2
Irradiated ALG / ECM				NS	NS	NS	NS
ALG / Col				NS	NS	NS	NS
ALG / ECM				NS	NS	NS	NS
ALG / ECM HSA					NS	NS	NS
ALG / ECM HSA / VEGF / TGF-β3						NS	NS
ALG / ECM HSA / VEGF / BMP-2							NS
ALG / ECM HSA / TGF - β3 / BMP-2							

One way ANOVA (Tukeys) p ≤ 0.001 - **Stro-1+ Cells**

Residual Hydrogel and Proteoglycan	Irradiated ALG / ECM	ALG / Col	ALG / ECM	ALG / ECM HSA	ALG / ECM HSA / VEGF / PTHrP	ALG / ECM HSA / VEGF / VitD3	ALG / ECM HSA / TGF - β3 / PTHrP	ALG / ECM HSA / TGF - β3 / VitD3
Irradiated ALG / ECM				**	NS	NS	NS	***
ALG / Col				NS	NS	NS	NS	NS
ALG / ECM				NS	NS	NS	NS	NS
ALG / ECM HSA	NS	NS	NS		NS	**	NS	NS
ALG / ECM HSA / VEGF / PTHrP	NS	**	***	NS		*	NS	NS
ALG / ECM HSA / VEGF / VitD3	NS	***	***	**	NS		NS	***
ALG / ECM HSA / TGF - β3 / PTHrP	NS	**	***	*	NS	NS		*
ALG / ECM HSA / TGF - β3 / VitD3	*	***	***	***	NS	NS	NS	

No Cells - *One way ANOVA (Tukeys) p ≤ 0.001*

One way ANOVA (Tukeys) p ≤ 0.01 - **Stro-1+ Cells**

Collagen Deposition	Irradiated ALG / ECM	ALG / Col	ALG / ECM	ALG / ECM HSA	ALG / ECM HSA / VEGF / PTHrP	ALG / ECM HSA / VEGF / VitD3	ALG / ECM HSA / TGF - β3 / PTHrP	ALG / ECM HSA / TGF - β3 / VitD3
Irradiated ALG / ECM				NS	NS	NS	*	*
ALG / Col				NS	NS	NS	NS	NS
ALG / ECM				NS	NS	NS	*	*
ALG / ECM HSA	NS	NS	NS		NS	NS	NS	NS
ALG / ECM HSA / VEGF / PTHrP	**	*	**	NS		NS	NS	NS
ALG / ECM HSA / VEGF / VitD3	***	***	***	**	NS		NS	NS
ALG / ECM HSA / TGF - β3 / PTHrP	**	*	**	NS	NS	NS		NS
ALG / ECM HSA / TGF - β3 / VitD3	NS	NS	NS	NS	NS	NS	NS	

No Cells - *One way ANOVA (Tukeys) p ≤ 0.001*

One way ANOVA (Tukeys) p > 0.05 - **Stro-1+ Cells**

Tissue Invasion	Irradiated ALG / ECM	ALG / Col	ALG / ECM	ALG / ECM HSA	ALG / ECM HSA / VEGF / PTHrP	ALG / ECM HSA / VEGF / VitD3	ALG / ECM HSA / TGF - β3 / PTHrP	ALG / ECM HSA / TGF - β3 / VitD3
Irradiated ALG / ECM				NS	NS	NS	NS	NS
ALG / Col				NS	NS	NS	NS	NS
ALG / ECM				NS	NS	NS	NS	NS
ALG / ECM HSA	NS	NS	NS		NS	NS	NS	NS
ALG / ECM HSA / VEGF / PTHrP	NS	NS	NS	NS		NS	NS	NS
ALG / ECM HSA / VEGF / VitD3	NS	NS	NS	NS	NS		NS	NS
ALG / ECM HSA / TGF - β3 / PTHrP	NS	NS	NS	NS	NS	NS		NS
ALG / ECM HSA / TGF - β3 / VitD3	NS	NS	NS	NS	NS	NS	NS	

No Cells - *One way ANOVA (Tukeys) p ≤ 0.05*

One way ANOVA (Tukeys) p ≤ 0.001 - **Residual Hydrogel and Proteoglycan**

Trek Cells	Irradiated ALG / ECM	ALG / Col	ALG / ECM	ALG / ECM HSA	ALG / ECM HSA / VEGF / PTHrP	ALG / ECM HSA / VEGF / VitD3	ALG / ECM HSA / TGF - β3 / PTHrP	ALG / ECM HSA / TGF - β3 / VitD3
Irradiated ALG / ECM					NS	*	**	***
ALG / Col					NS	NS	NS	NS
ALG / ECM					NS	NS	NS	*
ALG / ECM HSA					NS	NS	NS	NS
ALG / ECM HSA / VEGF / PTHrP	NS	NS	NS	NS		NS	NS	NS
ALG / ECM HSA / VEGF / VitD3	***	***	***	***	***		NS	NS
ALG / ECM HSA / TGF - β3 / PTHrP	***	***	***	***	***	NS		NS
ALG / ECM HSA / TGF - β3 / VitD3	NS	NS	NS	NS	NS	***	***	

Collagen Deposition - *One way ANOVA (Tukeys) p ≤ 0.001*

One way ANOVA (Tukeys) p ≤ 0.01 - **Tissue Invasion**

Trek Cells	Irradiated ALG / ECM	ALG / Col	ALG / ECM	ALG / ECM HSA	ALG / ECM HSA / VEGF / PTHrP	ALG / ECM HSA / VEGF / VitD3	ALG / ECM HSA / TGF - β3 / PTHrP	ALG / ECM HSA / TGF - β3 / VitD3
Irradiated ALG / ECM					NS	NS	NS	**
ALG / Col					NS	NS	NS	NS
ALG / ECM					NS	NS	NS	**
ALG / ECM HSA					NS	NS	NS	*
ALG / ECM HSA / VEGF / PTHrP						NS	NS	NS
ALG / ECM HSA / VEGF / VitD3							NS	NS
ALG / ECM HSA / TGF - β3 / PTHrP								NS
ALG / ECM HSA / TGF - β3 / VitD3								

Supplementary figure 6: Statistical analysis of histology data between growth factor groups from Alcian blue/Sirius red stained sections. Residual ECM proteoglycan and new proteoglycan deposition, collagen deposition, and tissue invasion were each statistically analysed. Comparison between all groups with Stro-1+ cells, without cells, or with TREK cells were assessed by a one-way ANOVA with Tukeys post-hoc test. For comparisons between control groups refer to Gothard D et al. 2015 [34]. NS indicates 'no significance.' * P ≤ 0.05, ** P ≤ 0.01, *** P ≤ 0.001.

ALG/ECM, alginate, and bone extracellular matrix; HSA, human serum albumin; VEGF, vascular endothelial growth factor; TGF-β_3, transforming growth factor beta 3; BMP-2, bone morphogenetic protein 2; PTHrP, parathyroid hormone-related protein; VitD3, vitamin D3.

A

One way ANOVA (Tukeys) p ≤ 0.01 - **Residual Hydrogel and Proteoglycan with Stro-1+ cells**

	Irradiated ALG / ECM	ALG / Col	ALG / ECM	ALG / ECM HSA	ALG / ECM HSA / VEGF / TGF-β3	ALG / ECM HSA / VEGF / BMP-2	ALG / ECM HSA / TGF-β3 / BMP-2
Irradiated ALG / ECM				*	NS	**	*
ALG / Col				NS	NS	NS	NS
ALG / ECM				NS	NS	NS	NS
ALG / ECM HSA	NS	NS	NS		NS	NS	NS
ALG / ECM HSA / VEGF / TGF-β3	*	NS	NS	NS		NS	NS
ALG / ECM HSA / VEGF / BMP-2	NS	NS	NS	NS	NS		NS
ALG / ECM HSA / TGF - β3 / BMP-2	*	NS	NS	NS	NS	NS	

Residual Hydrogel and Proteoglycan without cells - *One way ANOVA (Tukeys) p ≤ 0.01*

One way ANOVA (Tukeys) p ≤ 0.001 - **Mineralisation with Stro-1+ cells**

	Irradiated ALG / ECM	ALG / Col	ALG / ECM	ALG / ECM HSA	ALG / ECM HSA / VEGF / TGF-β3	ALG / ECM HSA / VEGF / BMP-2	ALG / ECM HSA / TGF-β3 / BMP-2
Irradiated ALG / ECM				**	***	NS	NS
ALG / Col				NS	NS	NS	NS
ALG / ECM				NS	NS	NS	NS
ALG / ECM HSA	NS	NS	NS		NS	NS	NS
ALG / ECM HSA / VEGF / TGF-β3	NS	*	***	NS		NS	NS
ALG / ECM HSA / VEGF / BMP-2	**	NS	NS	NS	**		NS
ALG / ECM HSA / TGF - β3 / BMP-2	NS	NS	*	NS	NS	*	

Mineralisation without cells - *One way ANOVA (Tukeys) p ≤ 0.001*

One way ANOVA (Tukeys) p > 0.05 - **Residual Hydrogel with TREK cells**

	Irradiated ALG / ECM	ALG / Col	ALG / ECM	ALG / ECM HSA	ALG / ECM HSA / VEGF / TGF-β3	ALG / ECM HSA / VEGF / BMP-2	ALG / ECM HSA / TGF-β3 / BMP-2
Irradiated ALG / ECM				NS	NS	NS	NS
ALG / Col				NS	NS	NS	NS
ALG / ECM				NS	NS	NS	NS
ALG / ECM HSA	*	NS	NS		NS	NS	NS
ALG / ECM HSA / VEGF / TGF-β3	NS	NS	NS	NS		NS	NS
ALG / ECM HSA / VEGF / BMP-2	*	NS	NS	NS	NS		NS
ALG / ECM HSA / TGF - β3 / BMP-2	**	NS	NS	NS	NS	NS	

Mineralisation with TREK cells - *One way ANOVA (Tukeys) p ≤ 0.001*

One way ANOVA (Tukeys) p ≤ 0.001 - **Residual Hydrogel and Proteoglycan with Stro-1+ cells**

	Irradiated ALG / ECM	ALG / Col	ALG / ECM	ALG / ECM HSA	ALG / ECM HSA / VEGF / PTHrP	ALG / ECM HSA / VEGF / VitD3	ALG / ECM HSA / TGF - β3 / PTHrP	ALG / ECM HSA / TGF - β3 / VitD3
Irradiated ALG / ECM				NS	**	NS	NS	***
ALG / Col				NS	NS	NS	NS	NS
ALG / ECM				NS	NS	NS	NS	*
ALG / ECM HSA	NS	NS	NS		NS	NS	NS	NS
ALG / ECM HSA / VEGF / PTHrP	***	**	***	**		*	NS	NS
ALG / ECM HSA / VEGF / VitD3	***	***	***	***	NS		NS	***
ALG / ECM HSA / TGF - β3 / PTHrP	***	*	**	*	NS	NS		*
ALG / ECM HSA / TGF - β3 / VitD3	***	***	***	***	NS	NS	NS	

Residual Hydrogel and Proteoglycan without cells - *One way ANOVA (Tukeys) p ≤ 0.001*

One way ANOVA (Tukeys) p ≤ 0.001 - **Mineralisation with Stro-1+ cells**

	Irradiated ALG / ECM	ALG / Col	ALG / ECM	ALG / ECM HSA	ALG / ECM HSA / VEGF / PTHrP	ALG / ECM HSA / VEGF / VitD3	ALG / ECM HSA / TGF - β3 / PTHrP	ALG / ECM HSA / TGF - β3 / VitD3
Irradiated ALG / ECM				*	NS	*	NS	NS
ALG / Col				NS	NS	NS	NS	**
ALG / ECM				NS	***	NS	*	***
ALG / ECM HSA	NS	NS	NS		NS	NS	NS	**
ALG / ECM HSA / VEGF / PTHrP	NS	***	***	NS		NS	NS	NS
ALG / ECM HSA / VEGF / VitD3	NS	***	***	**	NS		NS	**
ALG / ECM HSA / TGF - β3 / PTHrP	NS	***	***	*	NS	NS		*
ALG / ECM HSA / TGF - β3 / VitD3	*	***	***	***	NS	NS	NS	

Mineralisation without cells - *One way ANOVA (Tukeys) p ≤ 0.001*

One way ANOVA (Tukeys) p ≤ 0.001 - **Residual Hydrogel with TREK cells**

	Irradiated ALG / ECM	ALG / Col	ALG / ECM	ALG / ECM HSA	ALG / ECM HSA / VEGF / PTHrP	ALG / ECM HSA / VEGF / VitD3	ALG / ECM HSA / TGF - β3 / PTHrP	ALG / ECM HSA / TGF - β3 / VitD3
Irradiated ALG / ECM					NS	**	***	***
ALG / Col					NS	NS	NS	NS
ALG / ECM					NS	NS	NS	**
ALG / ECM HSA					NS	NS	*	**
ALG / ECM HSA / VEGF / PTHrP	NS	***	***	***		NS	NS	NS
ALG / ECM HSA / VEGF / VitD3	*	***	***	***	NS		NS	NS
ALG / ECM HSA / TGF - β3 / PTHrP	**	***	***	***	NS	NS		NS
ALG / ECM HSA / TGF - β3 / VitD3	NS	**	***	**	NS	NS	*	

Mineralisation with TREK cells - *One way ANOVA (Tukeys) p ≤ 0.001*

B

Intra-group comparisons and significance

		Residual Hydrogel and Proteoglycan		Mineralisation	
		One way ANOVA	Tukeys	One way ANOVA	Tukeys
ALG / ECM HSA	Stro-1+ cells / No cells	P ≤ 0.05	NS	P > 0.05	NS
	Stro-1+ cells / TREK		*		NS
	No cells / TREK		NS		NS
ALG / ECM HSA / VEGF / TGF-β3	Stro-1+ cells / No cells	P ≤ 0.001	**	P ≤ 0.001	***
	Stro-1+ cells / TREK		NS		*
	No cells / TREK		***		*
ALG / ECM HSA / VEGF / BMP-2	Stro-1+ cells / No cells	P ≤ 0.05	*	P > 0.05	NS
	Stro-1+ cells / TREK		NS		NS
	No cells / TREK		NS		NS
ALG / ECM HSA / TGF-β3 / BMP-2	Stro-1+ cells / No cells	P > 0.05	NS	P ≤ 0.001	*
	Stro-1+ cells / TREK		NS		NS
	No cells / TREK		NS		***

Intra-group comparisons and significance

		Residual Hydrogel and Proteoglycan		Mineralisation	
		One way ANOVA	Tukeys	One way ANOVA	Tukeys
ALG / ECM HSA	Stro-1+ cells / No cells	P > 0.05	NS	P > 0.05	NS
	Stro-1+ cells / TREK		NS		NS
	No cells / TREK		NS		NS
ALG / ECM HSA / VEGF / PTHrP	Stro-1+ cells / No cells	P > 0.05	NS	P ≤ 0.01	**
	Stro-1+ cells / TREK		NS		**
	No cells / TREK		NS		NS
ALG / ECM HSA / VEGF / VitD3	Stro-1+ cells / No cells	P ≤ 0.001	***	P ≤ 0.001	***
	Stro-1+ cells / TREK		***		***
	No cells / TREK		*		NS
ALG / ECM HSA / TGF-β3 / PTHrP	Stro-1+ cells / No cells	P ≤ 0.001	**	P ≤ 0.001	***
	Stro-1+ cells / TREK		**		***
	No cells / TREK		NS		NS
ALG / ECM HSA / TGF-β3 / VitD3	Stro-1+ cells / No cells	P > 0.05	NS	P ≤ 0.001	***
	Stro-1+ cells / TREK		NS		NS
	No cells / TREK		NS		***

Supplementary figure 7: Statistical analysis of torn/shredded areas within hydrogel samples following Alcian blue/Sirius red and Von Kossa stain. Comparison between all groups with Stro 1+ cells, without Stro-1+ cells, or with TREK cells were assessed by a one-way ANOVA with Tukeys post-hoc test (A). Statistically significant intra-group differences are shown in (B). NS indicates 'no significance,' * $P \leq 0.05$, ** $P \leq 0.01$, *** $P \leq 0.001$.

ALG/ECM, alginate, and bone extracellular matrix; HSA, human serum albumin; VEGF, vascular endothelial growth factor; TGF-β_3, transforming growth factor beta 3; BMP-2, bone morphogenetic protein 2; PTHrP, parathyroid hormone-related protein; VitD3, vitamin D3.

One way ANOVA (Tukeys) - **Intra-group significance**

		Residual Hydrogel and Proteoglycan		Collagen Deposition		Tissue Invasion	
		One way ANOVA	Tukeys	One way ANOVA	Tukeys	One way ANOVA	Tukeys
ALG / ECM HSA	Stro-1+ cells / No cells		NS		NS		NS
	Stro-1+ cells / TREK	P ≤ 0.05	NS	P > 0.05	NS	P ≤ 0.01	NS
	No cells / TREK		NS		NS		**
ALG / ECM HSA / VEGF / TGF-β3	Stro-1+ cells / No cells		NS		***		NS
	Stro-1+ cells / TREK	P ≤ 0.001	**	P ≤ 0.001	NS	P ≤ 0.05	NS
	No cells / TREK		**		***		*
ALG / ECM HSA / VEGF / BMP-2	Stro-1+ cells / No cells		NS		*		NS
	Stro-1+ cells / TREK	P > 0.05	NS	P ≤ 0.05	NS	P ≤ 0.05	NS
	No cells / TREK		NS		NS		NS
ALG / ECM HSA / TGF-β3 / BMP-2	Stro-1+ cells / No cells		NS		NS		NS
	Stro-1+ cells / TREK	P > 0.05	NS	P > 0.05	NS	P ≤ 0.01	***
	No cells / TREK		NS		NS		NS

One way ANOVA (Tukeys) - **Intra-group significance continued**

		Mineralisation		Cell Invasion		Vasculature	
		One way ANOVA	Tukeys	One way ANOVA	Tukeys	One way ANOVA	Tukeys
ALG / ECM HSA	Stro-1+ cells / No cells		NS		NS		NS
	Stro-1+ cells / TREK	P > 0.05	NS	P ≤ 0.01	***	P > 0.05	NS
	No cells / TREK		NS		***		NS
ALG / ECM HSA / VEGF / TGF-β3	Stro-1+ cells / No cells		***		NS		*
	Stro-1+ cells / TREK	P ≤ 0.001	NS	P ≤ 0.05	NS	P ≤ 0.05	NS
	No cells / TREK		**		*		NS
ALG / ECM HSA / VEGF / BMP-2	Stro-1+ cells / No cells		NS		NS		NS
	Stro-1+ cells / TREK	P > 0.05	NS	P ≤ 0.01	**	P > 0.05	NS
	No cells / TREK		NS		**		NS
ALG / ECM HSA / TGF-β3 / BMP-2	Stro-1+ cells / No cells		*		NS		NS
	Stro-1+ cells / TREK	P ≤ 0.001	***	P > 0.05	NS	P > 0.05	NS
	No cells / TREK		NS		NS		NS

One way ANOVA (Tukeys) - **Intra-group significance**

		Residual Hydrogel and Proteoglycan		Collagen Deposition		Tissue Invasion	
		One way ANOVA	Tukeys	One way ANOVA	Tukeys	One way ANOVA	Tukeys
ALG / ECM HSA	Stro-1+ cells / No cells		*		NS		NS
	Stro-1+ cells / TREK	P ≤ 0.05	NS	P > 0.05	NS	P > 0.05	NS
	No cells / TREK		NS		NS		NS
ALG / ECM HSA / VEGF / PTHrP	Stro-1+ cells / No cells		NS		*		NS
	Stro-1+ cells / TREK	P ≤ 0.05	NS	P ≤ 0.05	NS	P > 0.05	NS
	No cells / TREK		*		NS		NS
ALG / ECM HSA / VEGF / VitD3	Stro-1+ cells / No cells		***		*		**
	Stro-1+ cells / TREK	P ≤ 0.001	***	P ≤ 0.001	***	P ≤ 0.01	NS
	No cells / TREK		*		NS		*
ALG / ECM HSA / TGF-β3 / PTHrP	Stro-1+ cells / No cells		**		NS		NS
	Stro-1+ cells / TREK	P ≤ 0.001	**	P ≤ 0.01	**	P > 0.05	NS
	No cells / TREK		NS		*		NS
ALG / ECM HSA / TGF-β3 / VitD3	Stro-1+ cells / No cells		NS		NS		NS
	Stro-1+ cells / TREK	P > 0.05	NS	P > 0.05	NS	P > 0.05	NS
	No cells / TREK		NS		NS		NS

One way ANOVA (Tukeys) - **Intra-group significance continued**

		Mineralisation		Cell Invasion		Vasculature	
		One way ANOVA	Tukeys	One way ANOVA	Tukeys	One way ANOVA	Tukeys
ALG / ECM HSA	Stro-1+ cells / No cells		NS		NS		NS
	Stro-1+ cells / TREK	P > 0.05	NS	P > 0.05	NS	P > 0.05	NS
	No cells / TREK		NS		NS		NS
ALG / ECM HSA / VEGF / PTHrP	Stro-1+ cells / No cells		**		NS		NS
	Stro-1+ cells / TREK	P ≤ 0.001	***	P ≤ 0.05	NS	P ≤ 0.01	**
	No cells / TREK		NS		*		NS
ALG / ECM HSA / VEGF / VitD3	Stro-1+ cells / No cells		***		NS		*
	Stro-1+ cells / TREK	P ≤ 0.001	***	P ≤ 0.05	NS	P ≤ 0.001	***
	No cells / TREK		NS		*		NS
ALG / ECM HSA / TGF-β3 / PTHrP	Stro-1+ cells / No cells		***		NS		NS
	Stro-1+ cells / TREK	P ≤ 0.001	***	P > 0.05	NS	P ≤ 0.05	**
	No cells / TREK		NS		NS		NS
ALG / ECM HSA / TGF-β3 / VitD3	Stro-1+ cells / No cells		***		**		NS
	Stro-1+ cells / TREK	P ≤ 0.001	NS	P ≤ 0.01	NS	P > 0.05	NS
	No cells / TREK		***		**		NS

Supplementary figure 8: Statistical analysis of histological data between those groups with Stro-1+ cell, without cell, and with TREK cell incorporation. NS indicates 'no significance.' * $P \leq 0.05$, ** $P \leq 0.01$, *** $P \leq 0.001$.

ALG/ECM, alginate, and bone extracellular matrix; HSA, human serum albumin; VEGF, vascular endothelial growth factor; TGF-β_3, transforming growth factor beta 3; BMP-2, bone morphogenetic protein 2; PTHrP, parathyroid hormone-related protein; VitD3, vitamin D3.

One way ANOVA (Tukeys) $p \leq 0.001$ - **Mineralisation with Stro-1+ cells**

	Irradiated ALG / ECM	ALG / Col	ALG / ECM	ALG / ECM HSA	ALG / ECM HSA / VEGF / TGF-β3	ALG / ECM HSA / VEGF / BMP-2	ALG / ECM HSA / TGF-β3 / BMP-2
**Irradiated ALG / ECM**				**	***	NS	NS
**ALG / Col**				NS	NS	NS	NS
**ALG / ECM**				NS	NS	*	***
**ALG / ECM HSA**	NS	NS	NS		NS	NS	NS
**ALG / ECM HSA / VEGF / TGF-β3**	NS	NS	NS	NS		NS	*
**ALG / ECM HSA / VEGF / BMP-2**	NS	NS	NS	NS	NS		NS
**ALG / ECM HSA / TGF - β3 / BMP-2**	**	NS	**	**	NS	*	

Cell Invasion with Stro-1+ cells - *One way ANOVA (Tukeys) $p \leq 0.01$*

One way ANOVA (Tukeys) $p \leq 0.001$ - **Mineralisation without cells**

	Irradiated ALG / ECM	ALG / Col	ALG / ECM	ALG / ECM HSA	ALG / ECM HSA / VEGF / TGF-β3	ALG / ECM HSA / VEGF / BMP-2	ALG / ECM HSA / TGF-β3 / BMP-2
**Irradiated ALG / ECM**				NS	NS	NS	NS
**ALG / Col**				NS	NS	NS	NS
**ALG / ECM**				NS	***	NS	NS
**ALG / ECM HSA**	NS	NS	NS		NS	NS	NS
**ALG / ECM HSA / VEGF / TGF-β3**	NS	NS	NS	NS		NS	NS
**ALG / ECM HSA / VEGF / BMP-2**	NS	NS	NS	NS	NS		NS
**ALG / ECM HSA / TGF - β3 / BMP-2**	NS	NS	NS	NS	NS	NS	

Cell Invasion without cells - *One way ANOVA (Tukeys) $p > 0.05$*

One way ANOVA (Tukeys) $p \leq 0.001$ - **Mineralisation with TREK cells**

	Irradiated ALG / ECM	ALG / Col	ALG / ECM	ALG / ECM HSA	ALG / ECM HSA / VEGF / TGF-β3	ALG / ECM HSA / VEGF / BMP-2	ALG / ECM HSA / TGF-β3 / BMP-2
**Irradiated ALG / ECM**				*	*	*	***
**ALG / Col**				NS	NS	NS	NS
**ALG / ECM**				NS	**	NS	NS
**ALG / ECM HSA**	NS	NS	NS		NS	NS	NS
**ALG / ECM HSA / VEGF / TGF-β3**	***	*	***	NS		NS	NS
**ALG / ECM HSA / VEGF / BMP-2**	**	NS	**	NS	NS		NS
**ALG / ECM HSA / TGF - β3 / BMP-2**	NS	NS	NS	NS	NS	NS	

Cell Invasion with TREK cells - *One way ANOVA (Tukeys) $p \leq 0.001$*

One way ANOVA (Tukeys) $p \leq 0.001$ - **Mineralisation with Stro-1+ cells**

	Irradiated ALG / ECM	ALG / Col	ALG / ECM	ALG / ECM HSA	ALG / ECM HSA / VEGF / PTHrP	ALG / ECM HSA / VEGF / VitD3	ALG / ECM HSA / TGF - β3 / PTHrP	ALG / ECM HSA / TGF - β3 / VitD3
**Irradiated ALG / ECM**				**	NS	***	*	NS
**ALG / Col**				NS	NS	NS	NS	NS
**ALG / ECM**				NS	***	NS	**	***
**ALG / ECM HSA**	NS	NS	NS		NS	NS	NS	**
**ALG / ECM HSA / VEGF / PTHrP**	**	NS	**	NS		NS	NS	NS
**ALG / ECM HSA / VEGF / VitD3**	NS	NS	NS	NS	NS		NS	***
**ALG / ECM HSA / TGF - β3 / PTHrP**	NS	NS	NS	NS	NS	NS		*
**ALG / ECM HSA / TGF - β3 / VitD3**	NS	NS	NS	NS	NS	NS	NS	

Cell Invasion with Stro-1+ cells - *One way ANOVA (Tukeys) $p \leq 0.01$*

One way ANOVA (Tukeys) $p \leq 0.001$ - **Mineralisation without cells**

	Irradiated ALG / ECM	ALG / Col	ALG / ECM	ALG / ECM HSA	ALG / ECM HSA / VEGF / PTHrP	ALG / ECM HSA / VEGF / VitD3	ALG / ECM HSA / TGF - β3 / PTHrP	ALG / ECM HSA / TGF - β3 / VitD3
**Irradiated ALG / ECM**				NS	NS	NS	NS	*
**ALG / Col**				NS	**	***	**	***
**ALG / ECM**				NS	***	***	***	***
**ALG / ECM HSA**	NS	NS	NS		NS	**	*	***
**ALG / ECM HSA / VEGF / PTHrP**	***	***	***	*		NS	NS	NS
**ALG / ECM HSA / VEGF / VitD3**	NS	NS	NS	NS	*		NS	NS
**ALG / ECM HSA / TGF - β3 / PTHrP**	NS	NS	NS	NS	**	NS		NS
**ALG / ECM HSA / TGF - β3 / VitD3**	**	**	***	NS	NS	NS	NS	

Cell Invasion without cells - *One way ANOVA (Tukeys) $p \leq 0.001$*

One way ANOVA (Tukeys) $p \leq 0.001$ - **Mineralisation with TREK cells**

	Irradiated ALG / ECM	ALG / Col	ALG / ECM	ALG / ECM HSA	ALG / ECM HSA / VEGF / PTHrP	ALG / ECM HSA / VEGF / VitD3	ALG / ECM HSA / TGF - β3 / PTHrP	ALG / ECM HSA / TGF - β3 / VitD3
**Irradiated ALG / ECM**					NS	**	*	NS
**ALG / Col**					NS	NS	***	NS
**ALG / ECM**					***	***	***	***
**ALG / ECM HSA**					***	***	***	*
**ALG / ECM HSA / VEGF / PTHrP**	NS	NS	NS	NS		NS	NS	NS
**ALG / ECM HSA / VEGF / VitD3**	NS	NS	NS	NS	NS		NS	*
**ALG / ECM HSA / TGF - β3 / PTHrP**	NS	NS	NS	NS	NS	NS		**
**ALG / ECM HSA / TGF - β3 / VitD3**	NS	NS	NS	NS	NS	NS	NS	

Cell Invasion with TREK cells - *One way ANOVA (Tukeys) $p > 0.05$*

Supplementary figure 9: Statistical analysis of histology data between growth factor groups from Von Kossa-stained sections. Mineralisation and cell invasion were both statistically analysed. Comparison between all groups with Stro-1+ cells, without cells, or with TREK cells were assessed by a one-way ANOVA with Tukeys post-hoc test. For comparisons between control groups refer to Gothard D et al. 2015 [34]. NS indicates 'no significance.' * $P \leq 0.05$, ** $P \leq 0.01$, *** $P \leq 0.001$.

ALG/ECM, alginate, and bone extracellular matrix; HSA, human serum albumin; VEGF, vascular endothelial growth factor; TGF-β_3, transforming growth factor beta 3; BMP-2, bone morphogenetic protein 2; PTHrP, parathyroid hormone-related protein; VitD3, vitamin D3.

One way ANOVA (Tukeys) p ≤ 0.001 - **Vascularisation with Stro-1+ cells**

	Irradiated ALG / ECM	ALG / Col	ALG / ECM	ALG / ECM HSA	ALG / ECM HSA / VEGF / TGF-β3	ALG / ECM HSA / VEGF / BMP-2	ALG / ECM HSA / TGF-β3 / BMP-2
Irradiated ALG / ECM				NS	***	*	NS
ALG / Col				NS	***	**	NS
ALG / ECM				NS	*	NS	NS
ALG / ECM HSA	NS	*	NS		NS	NS	NS
ALG / ECM HSA / VEGF / TGF-β3	NS	NS	NS	NS		NS	*
ALG / ECM HSA / VEGF / BMP-2	***	***	**	NS	*		NS
ALG / ECM HSA / TGF - β3 / BMP-2	NS	NS	NS	NS	NS	**	

Vascularisation without cells - *One way ANOVA (Tukeys) p ≤ 0.001*

One way ANOVA (Tukeys) p ≤ 0.01 - **Vascularisation with TREK cells**

	Irradiated ALG / ECM	ALG / Col	ALG / ECM	ALG / ECM HSA	ALG / ECM HSA / VEGF / TGF-β3	ALG / ECM HSA / VEGF / BMP-2	ALG / ECM HSA / TGF-β3 / BMP-2
Irradiated ALG / ECM				NS	*	*	NS
ALG / Col				NS	*	*	NS
ALG / ECM				NS	NS	NS	NS
ALG / ECM HSA					NS	NS	NS
ALG / ECM HSA / VEGF / TGF-β3						NS	NS
ALG / ECM HSA / VEGF / BMP-2							NS
ALG / ECM HSA / TGF - β3 / BMP-2							

One way ANOVA (Tukeys) p ≤ 0.001 - **Vascularisation with Stro-1+ cells**

	Irradiated ALG / ECM	ALG / Col	ALG / ECM	ALG / ECM HSA	ALG / ECM HSA / VEGF / PTHrP	ALG / ECM HSA / VEGF / VitD3	ALG / ECM HSA / TGF - β3 / PTHrP	ALG / ECM HSA / TGF - β3 / VitD3
Irradiated ALG / ECM				NS	**	**	***	**
ALG / Col				NS	**	**	***	**
ALG / ECM				NS	NS	NS	***	NS
ALG / ECM HSA	NS	NS	NS		NS	NS	**	NS
ALG / ECM HSA / VEGF / PTHrP	NS	*	NS	NS		NS	NS	NS
ALG / ECM HSA / VEGF / VitD3	NS	NS	NS	NS	NS		NS	NS
ALG / ECM HSA / TGF - β3 / PTHrP	*	**	NS	NS	NS	NS		NS
ALG / ECM HSA / TGF - β3 / VitD3	NS	NS	NS	NS	NS	NS	NS	

Vascularisation without cells - *One way ANOVA (Tukeys) p ≤ 0.001*

One way ANOVA (Tukeys) p > 0.05 - **Vascularisation with TREK cells**

	Irradiated ALG / ECM	ALG / Col	ALG / ECM	ALG / ECM HSA	ALG / ECM HSA / VEGF / PTHrP	ALG / ECM HSA / VEGF / VitD3	ALG / ECM HSA / TGF - β3 / PTHrP	ALG / ECM HSA / TGF - β3 / VitD3
Irradiated ALG / ECM				NS	NS	NS	NS	NS
ALG / Col				NS	NS	NS	NS	NS
ALG / ECM				NS	NS	NS	NS	NS
ALG / ECM HSA					NS	NS	NS	NS
ALG / ECM HSA / VEGF / PTHrP						NS	NS	NS
ALG / ECM HSA / VEGF / VitD3							NS	NS
ALG / ECM HSA / TGF - β3 / PTHrP								NS
ALG / ECM HSA / TGF - β3 / VitD3								

Supplementary figure 10: Statistical analysis of hydrogels stained with Goldner's Trichrome. Vascularisation was statistically analysed via the presence of red blood cells. Comparison between all groups with Stro-1+ cells, without Stro-1+ cells or with TREK cells were assessed by a one-way ANOVA with Tukeys post-hoc test. For comparisons between control groups refer to Gothard D et al. 2015 [34]. NS indicates 'no significance.' * P ≤ 0.05, ** P ≤ 0.01, *** P ≤ 0.001.

ALG/ECM, alginate, and bone extracellular matrix; HSA, human serum albumin; VEGF, vascular endothelial growth factor; TGF-β_3, transforming growth factor beta 3; BMP-2, bone morphogenetic protein 2; PTHrP, parathyroid hormone-related protein; VitD3, vitamin D3.

A

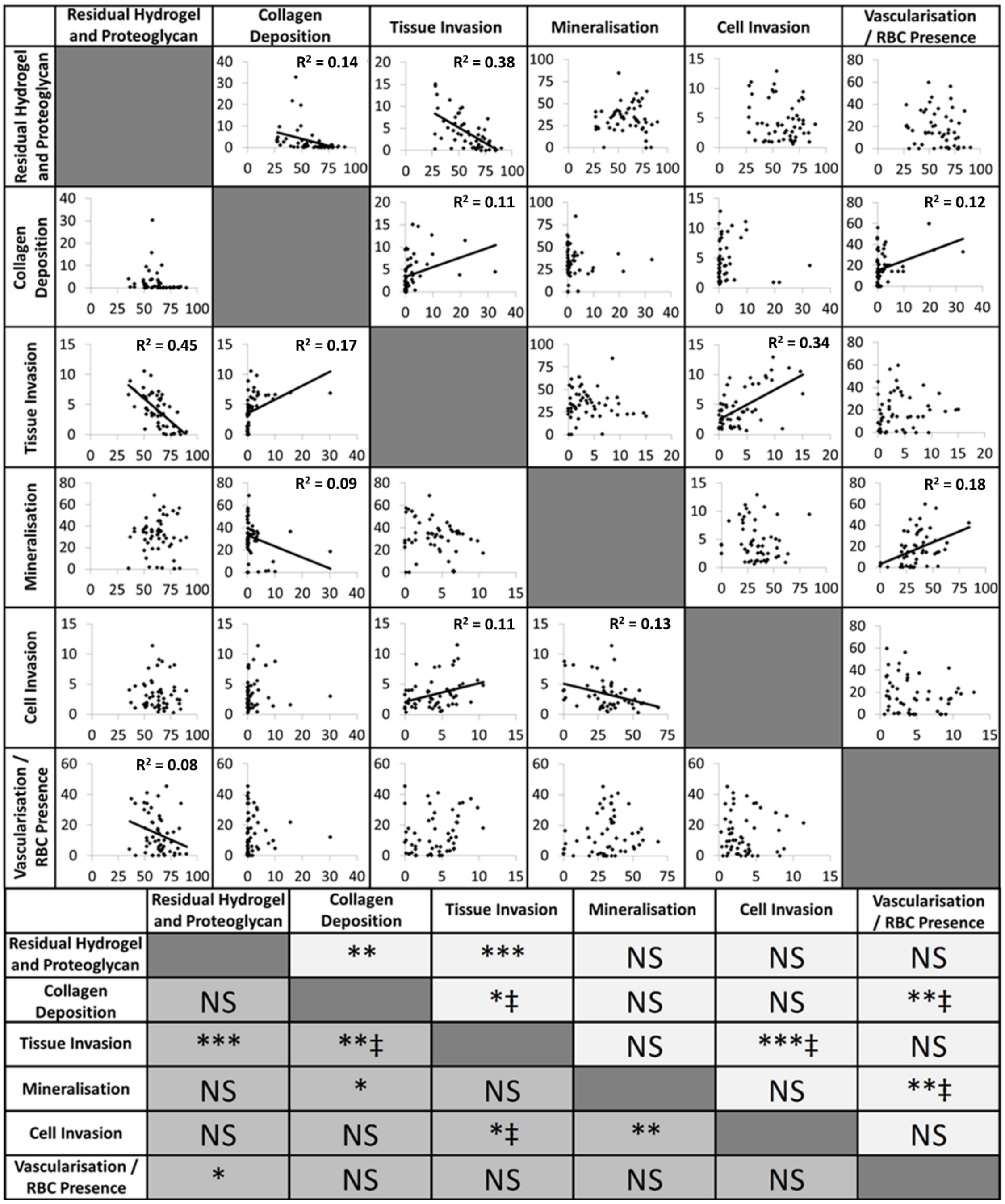

	Residual Hydrogel and Proteoglycan	Collagen Deposition	Tissue Invasion	Mineralisation	Cell Invasion	Vascularisation / RBC Presence
Residual Hydrogel and Proteoglycan		**	***	NS	NS	NS
Collagen Deposition	NS		*‡	NS	NS	**‡
Tissue Invasion	***	**‡		NS	***‡	NS
Mineralisation	NS	*	NS		NS	**‡
Cell Invasion	NS	NS	*‡	**		NS
Vascularisation / RBC Presence	*	NS	NS	NS	NS	

B

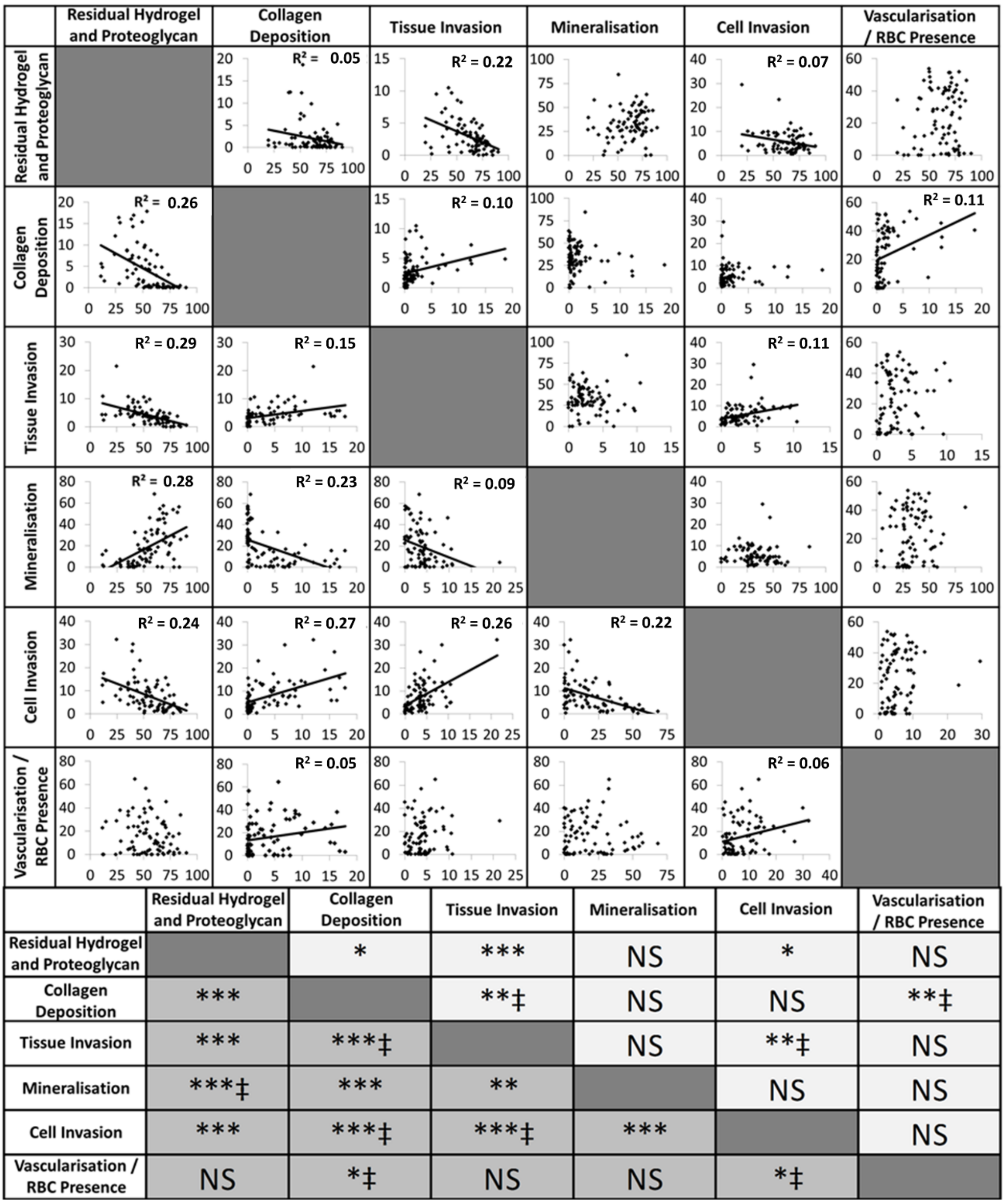

	Residual Hydrogel and Proteoglycan	Collagen Deposition	Tissue Invasion	Mineralisation	Cell Invasion	Vascularisation / RBC Presence
Residual Hydrogel and Proteoglycan		*	***	NS	*	NS
Collagen Deposition	***		**‡	NS	NS	**‡
Tissue Invasion	***	***‡		NS	**‡	NS
Mineralisation	***‡	***	**		NS	NS
Cell Invasion	***	***‡	***‡	***		NS
Vascularisation / RBC Presence	NS	*‡	NS	NS	*‡	

Supplementary figure 11: Statistical analysis of correlations between histologically assessed parameters within groups with and without Stro-1+ cell incorporation. Following colour quantification of A/S, VK and GT stained samples, data for residual hydrogel and proteoglycan deposition, collagen deposition, tissue invasion, mineralisation, cell invasion, and vascularisation (RBC presence) were combined across all growth factor groups and correlations between colours were assessed. Top right graphs depict correlations between parameters from

all groups with Stro-1+ cell incorporation. Bottom left graphs depict correlations between parameters from all groups without Stro-1+ cell incorporation. Trend lines highlight those graphs depicting significant correlations. Statistical analysis is shown in table format beneath the charts. NS indicates 'no significance.' ‡ denotes positive correlation. * $P \leq 0.05$, ** $P \leq 0.01$, *** $P \leq 0.001$.

A/S, Alcian Blue / Sirius Red; VK, Von Kossa; GT, Goldners Trichrome.

A

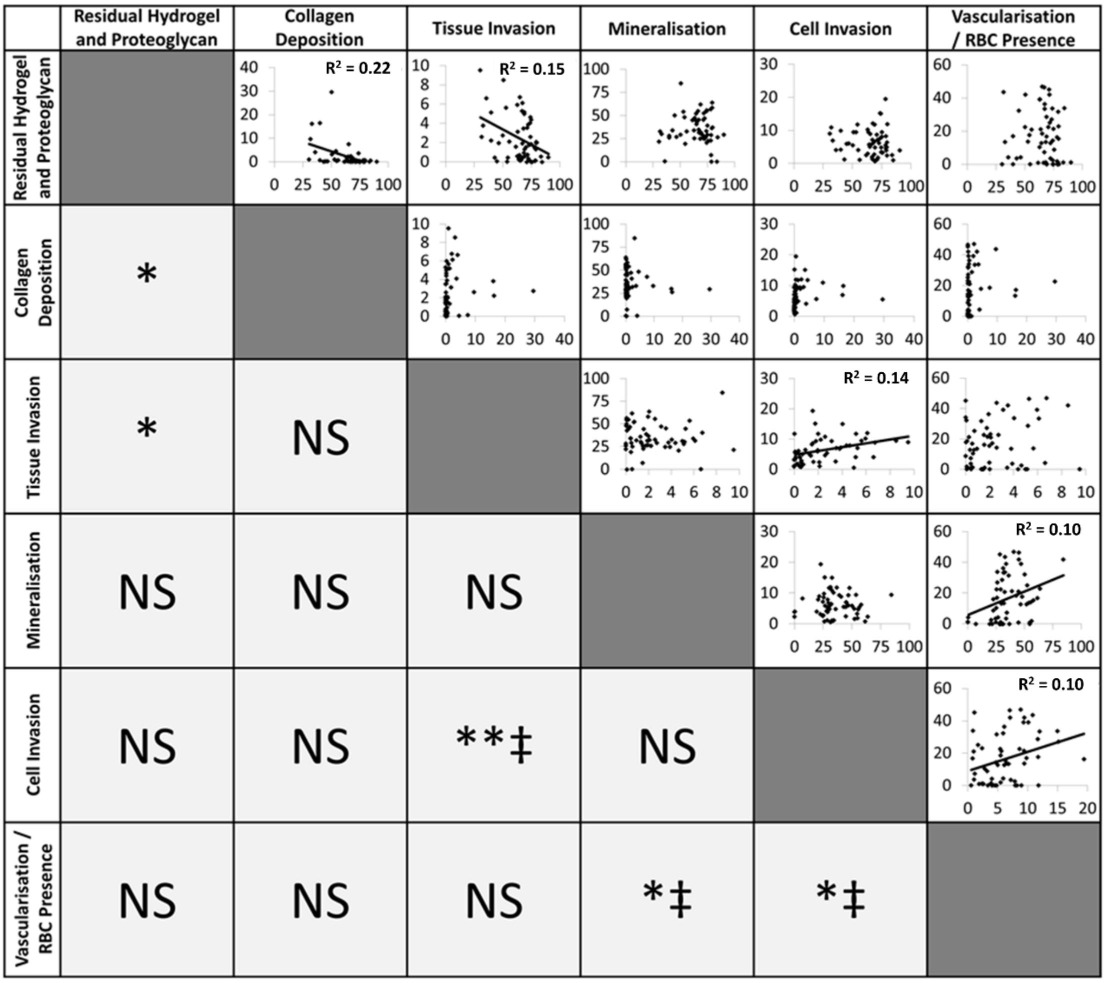
Residual Hydrogel and Proteoglycan
Collagen Deposition
Tissue Invasion
Mineralisation
Cell Invasion
Vascularisation / RBC Presence
R² = 0.22
R² = 0.15
R² = 0.14
R² = 0.10
R² = 0.10
*
*
NS
NS
NS
NS
NS
NS
**‡
NS
NS
NS
NS
*‡
*‡

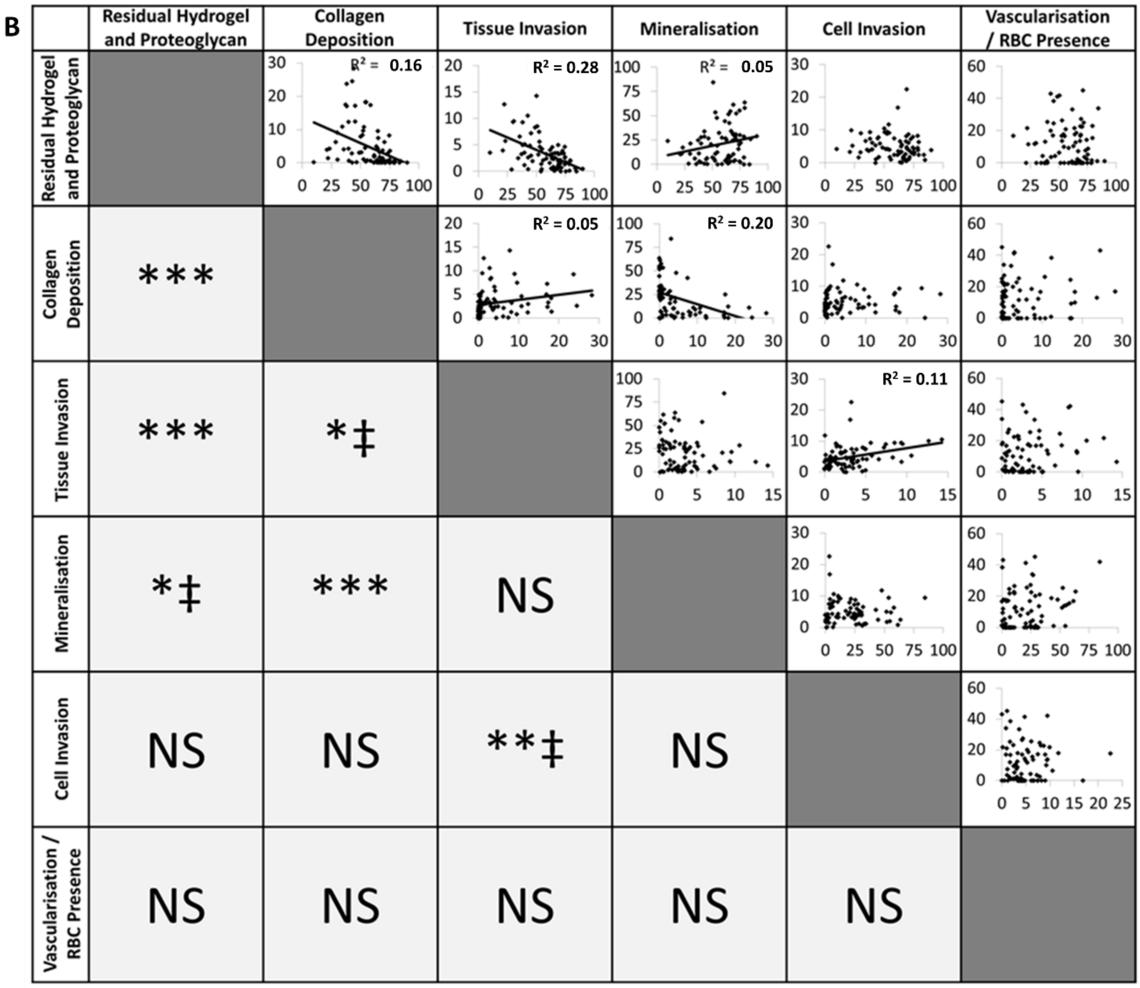

Supplementary figure 12: Statistical analysis of correlations between histologically assessed parameters within groups under mechanotransduction. Following colour quantification of A/S, VK and GT stained samples, data for residual hydrogel and proteoglycan deposition, collagen deposition, tissue invasion, mineralisation, cell invasion and vascularisation (RBC presence) was combined across all growth factor groups and correlations between colours were assessed. Top right graphs depict correlations between parameters from all groups with Stro-1+ cell incorporation and mechanotransduction. Bottom left table depicts statistical analysis between parameters. Trend lines highlight those graphs depicting significant correlations. NS indicates 'no significance.' ‡ denotes positive correlation. * P ≤ 0.05, ** P ≤ 0.01, *** P ≤ 0.001.

A/S, Alcian Blue / Sirius Red; VK, Von Kossa; GT, Goldners Trichrome.
